# Characteristics of *Epicoccum latusicollum* as revealed by genomic and metabolic phenomic analysis, the causal agent of tobacco *Epicoccus* leaf spot

**DOI:** 10.3389/fpls.2023.1199956

**Published:** 2023-08-24

**Authors:** Zhen Li, Jing-rong Hu, Wen-hong Li, Han-cheng Wang, Zhen-ni Guo, Xing Cheng, Liu-ti Cai, Cai-hua Shi

**Affiliations:** ^1^ College of Agriculture, Yangtze University, Jingzhou, Hubei, China; ^2^ Guizhou Provincial Academician Workstation of Microbiology and Health, Guizhou Academy of Tobacco Science, Guiyang, Guizhou, China; ^3^ Institute of Advanced Agricultural Science, Hubei University of Arts and Science, Xiangyang, Hubei, China; ^4^ Guizhou Institute of Plant Protection, Guizhou Academy of Agricultural Sciences, Guiyang, Guizhou, China; ^5^ MGI Tech Co., Ltd Research and Development Centre for Laboratory Automation, Shenzhen, Guangzhou, China; ^6^ College of Ecology and Environment, Hainan University, Haikou, Hainan, China; ^7^ School of Food Science and Technology & School of Chemical Engineering, Hubei University of Arts and Science, Xiangyang, Hubei, China

**Keywords:** flue-cured tobacco, *Epicoccum latusicollum*, biological characteristics, whole genome sequence, Biolog Phenotype MicroArray

## Abstract

*Epicoccum latusicollum* is a fungus that causes a severe foliar disease on flue-cured tobacco in southwest China, resulting in significant losses in tobacco yield and quality. To better understand the organism, researchers investigated its optimal growth conditions and metabolic versatility using a combination of traditional methods and the Biolog Phenotype MicroArray technique. The study found that *E. latusicollum* exhibited impressive metabolic versatility, being able to metabolize a majority of carbon, nitrogen, sulfur, and phosphorus sources tested, as well as adapt to different environmental conditions, including broad pH ranges and various osmolytes. The optimal medium for mycelial growth was alkyl ester agar medium, while oatmeal agar medium was optimal for sporulation, and the optimum temperature for mycelial growth was 25°C. The lethal temperature was 40°C. The study also identified arbutin and amygdalin as optimal carbon sources and Ala-Asp and Ala-Glu as optimal nitrogen sources for *E. latusicollum*. Furthermore, the genome of *E. latusicollum* strain T41 was sequenced using Illumina HiSeq and Pacific Biosciences technologies, with 10,821 genes predicted using Nonredundant, Gene Ontology, Clusters of Orthologous Groups, Kyoto Encyclopedia of Genes and Genomes, and SWISS-PROT databases. Analysis of the metabolic functions of phyllosphere microorganisms on diseased tobacco leaves affected by *E. latusicollum* using the Biolog Eco microplate revealed an inability to efficiently metabolize a total of 29 carbon sources, with only tween 40 showing some metabolizing ability. The study provides new insights into the structure and function of phyllosphere microbiota and highlights important challenges for future research, as well as a theoretical basis for the integrated control and breeding for disease resistance of tobacco *Epicoccus* leaf spot. This information can be useful in developing new strategies for disease control and management, as well as enhancing crop productivity and quality.

## 1 Introduction

Tobacco (*Nicotiana tabacum* L.) is a commercial leafy annual solanaceous plant grown for its leaves (Chen et al., 2020), and is widely cultivated in Guizhou, China, with an average annual cultivation area of over 120,000 hectares in the past 5 years (Yan et al., 2021). It is a significant source of income for farmers and enriches the local agricultural industry (Yan et al., 2016). However, leaf spot caused by *Epicoccum latusicollum* has been found in tobacco-producing areas (Guo et al., 2021), with symptoms including sandy beige, elliptical, or irregular-shaped lesions with a brown edge, surrounded by yellow halos, affecting approximately 40% of leaves on 5% of plants. This fungal genus has a wide host range and can infect various plants such as *Lippia multiflora* (Huang et al., 2021a), *Sorghum* (Stokholm et al., 2016), *Platostoma palustre* (Khoo et al., 2022a), *Paeonia suffruticosa* (Yang et al., 2022), *Basella alba* (Khoo et al., 2022b), and *Oxalis corymbosa* (Niu et al., 2022).

Molecular biology advancements allowed for the reclassification of certain species within the family Didymellaceae, transferring them to the newly established genus *Epicoccum* (Aveskamp et al., 2010). Subsequent studies by Chen et al. (2017) refined the taxonomy of *Epicoccum* and introduced additional species, including *E. latusicollum*, *E. viticis*, and *E. duchesneae*, using molecular markers and phylogenetic analysis and the pathogenesis of *E. latusicollum* is poorly understood. It has been reported to infect various commercial crops, such as *Dioscorea sativa* (Han et al., 2019), *Zea mays* (Xu et al., 2022), and *Elaeagnus pungens* (Qi et al., 2021), as well as *Nicotiana tabacum*, preliminarily explore the pathogen and biological characteristics on tobacco (He et al., 2022). Current research on *E. latusicollum* has focused primarily on its occurrence and identification, with less attention paid to its biological characteristics. In particular, there has been a lack of investigation into the phenotypic characteristics of *E. latusicollum*, including its utilization of different substrates. Therefore, the potential functional roles of this microorganism in the environment remain largely unknown. A promising approach is the use of the Biolog PM system, which can provide a comprehensive profile of the metabolic capabilities of this microorganism. By identifying the specific substrates that *E. latusicollum* is able to utilize, we can gain insight into its potential functional roles and ecological significance. This study investigated the biological characteristics of *E. latusicollum*, such as temperature, medium, and light conditions, and to explore its carbon, nitrogen, sulfur, and phosphorus source utilization, and the effects of osmotic, ionic, and pH environments using the Biolog PM system, this system can detect nearly 900 metabolic phenotypes at one time (Bochner et al., 2001; Bochner, 2003). A better understanding of the phenotypic characters of the pathogen can be valuable in developing management practices to control the disease below economic thresholds (Wang et al., 2015).To date, the genomes of many fungi have been published in the National Center for Biotechnology Information (NCBI: https://ncbi.nlm.nih.gov/), based on the available genome sequences, researchers have determined the evolutionary relationships of many fungi. The study mentioned by Zhang et al. (2018) aimed to investigate the pathogenesis, growing characteristics, and molecular mechanisms of fungal development, metabolism, systematic taxonomy, and evolution at the molecular level. To achieve these, the study utilized whole genome sequencing (WGS) to obtain a comprehensive profile of the genetic makeup of the fungus. The use of whole genome sequencing enabled the researchers to obtain a detailed understanding of the genetic basis of the fungus’s biology, paving the way for further research into the molecular mechanisms underlying fungal development and evolution. At the assembly level, the types of transposable elements and transcriptional factors (TFs) were further analyzed. This genomic resource will provide a new insight to better understand the relevance of phenotypic characters and genetic mechanisms in *E. latusicollum.*

One effective method for evaluating microbial indicators is to use community-level physiological profiles (CLPP) assessed through Biolog EcoPlates™. CLPP can detect multiple microbial metabolic activities, making it a powerful tool for investigating microbial communities (Liu et al., 2015). By comparing the patterns of physiological fingerprints between different samples, valuable information about microbial community diversity can be obtained (Crecchio et al., 2004). Following this approach, Insam (1997) proposed a 96-well microplate with 31 substrates plus control, each in three replications (EcoPlate) as a new set of substrates for community characterization in environmental samples. The CLPP method provides an exciting opportunity to overcome the drawbacks of alternative time consuming culture-based analyses or biochemical tests (Preston-Mafham et al., 2002; Schutter and Dick, 2001). Biolog-ECO metabolic phenotyping was used to analyze the metabolic diversity of the microbial community in healthy and diseased tobacco leaves affected by tobacco *Epicoccus* leaf spot, to clarify multiple microbial metabolic activities and investigate microbial community diversity. This analysis aimed to provide a basis for controlling the occurrence of tobacco *Epicoccus* leaf spot by optimizing the carbon supply structure of tobacco and improving the microbiota (Bochner, 1989; Insam, 1997).

Therefore, the objectives of this study were: (i) to investigate the basic biological characteristics and metabolic phenotype of *E. latusicollum*, (ii) to sequence the genome of *E. latusicollum*, and (iii) in order to elucidate the metabolic functions of phyllosphere microorganisms in tobacco affected by *Epicoccus* leaf spot and community level of physiological profiles. This study provides insights into the pathogenesis, biological characteristics, and genomics of *E. latusicollum*, a fungal pathogen that causes foliar diseases in tobacco-producing areas, outcome of this study may gain a better understanding of the genetic mechanisms and phenotypic characteristics of the pathogen, and may also provide some effective management suggestions to reduce the impact of the disease below economic thresholds.

## 2 Materials and methods

### 2.1 Strain materials and culture conditions

One isolate of the pathogenic *E. latusicollum* strain T41, randomly selected from multiple isolates, was analyzed in the laboratory of Guizhou Academy of Tobacco Science (Huang et al., 2021b). The strain (T41) of *E. latusicollum* was stored short-term in a 4°C refrigerator and long-term using cryopreservation. For cryopreservation, mycelial plugs were immersed in a cryoprotective solution containing glycerol and kept at -80°C, allowing for future revival by transferring to suitable growth media and identified as *E. latusicollum* by DNA sequence similarity (Guo et al., 2021). The growth rate and colony morphology of *E. latusicollum* T41 were determined at different temperatures and on different media. Additionally, the mycelial growth rate was assessed under different light and dark conditions. GenBank accession numbers MN704804, MN710367, MN718012, and MN718013 were assigned to the strain T41. Preliminary phylogenetic analyses of the genus *Epicoccum* were conducted using LSU, ITS, TUB, and *rpb2* gene sequences. A dataset comprising 68 available sequences, primarily sourced from Guo et al. (2021), was utilized. The Maximum parsimony (MP) algorithm proposed by Swofford & Sullivan was employed to identify the closely related strains. Sequences generated in this study were lodged in GenBank (**Supplementary Table 1**).

The present study aimed to investigate the impact of temperature on the growth of *E. latusicollum* T41, a filamentous fungus, by culturing it on various agar media, namely potato dextrose agar (PDA), alkyl ester agar (AEA), and oatmeal agar (OA), over a temperature range of 10°C to 40°C. To ensure the reliability of the findings, three replicate plates were used for each temperature, and adverse temperature tests were carried out to assess the strain’s ability to withstand unfavorable conditions. In cases where non-growing mycelia were observed, re-culturing was performed under optimal temperature conditions to verify the strain’s viability. The effect of light on *E. latusicollum* T41 growth was also investigated by culturing the strain on PDA at 25°C under continuous darkness, continuous illumination and alternation of light and darklight. The growth of the fungus was assessed by measuring the colony diameters after five days of incubation. To minimize any experimental bias, each treatment was conducted in triplicate, and the entire experiment was repeated three times to ensure consistency and reproducibility of the results.

### 2.2 Phenotype MicroArray assays

The metabolic capacity of *E. latusicollum* was assessed using the Phenotype MicroArray (PM) system (Biolog, Hayward, CA, USA) to determine its phenotype (Bochner et al., 2001; Zhou et al., 2003; Von Eiff et al., 2006). In this study, the Biolog PM system was employed to evaluate the catabolic pathways. To accomplish this, plates 1-8 were utilized to assess the utilization of different carbon, nitrogen, phosphorus, and sulfur sources, providing insights into the metabolic versatility of the organism. Additionally, plates 9-10 were used to investigate the effects of osmotic/ion and pH on the organism’s metabolic activities. The Biolog corporation was the source of all materials, media, and reagents employed in the PM system, ensuring the consistency of the experimental results.

To obtain the spores of the strain under investigation, OA medium was utilized as the growth and maintenance substrate in a climate-controlled cabinet at 25°C, under alternating light and dark conditions. After 15 days of incubation, conidia were generated. To collect the spores, sterile cotton swabs were moistened with FF Inoculating Fluid (FF-IF), and then rotated on the surface of the colony to absorb the spores. The swabs were then immersed in 12 mL of FF-IF inoculation solution, and the resulting suspension was filtered through a double layer of sterile cheesecloth with a Grade #40 and 24 × 20 threads per inch to eliminate mycelial fragments (Li et al., 2023). The resulting conidia suspension was subsequently diluted to a final concentration of 1×10^5^ spores mL^-1^ in a suitable medium containing sterile FF-IF, to ensure the absence of contaminants that could affect the accuracy and reliability of the experimental results.

One hundred microliters (μl) of a dilution of 62% transmittance (T) suspension of cells were added to each well of the PM plates. FF-IF was used for PM plates 1 and 2, FF-IF plus 100 mM D-glucose, 5 mM potassium phosphate (pH 6.0), and 2 mM sodium sulfate was used for PM plates 3 and 6–8. FF-IF plus 100 mM D-glucose was used for PM plate 4. FF-IF plus yeast nitrogen base and 100 mM D-glucose was used for PM plates 9 and 10 (Wang et al., 2015). These specific media formulations were employed to assess the microorganism’s utilization of carbon, nitrogen, phosphorus and sulfur sources under different environmental conditions. Plates containing 960 µL of the specified mixture were incubated in an OmniLog at 28°C for one week, and readings were taken every 15 minutes.

Phenotypic data were recorded by capturing digital images of microarrays and storing turbidity values. Kinetic and Parametric software (Biolog, Hayward, CA, USA) was used to analyze the data. The phenotype was estimated according to the area of each well under the staining formation kinetics curve (Vaas et al., 2012), and the experiment was repeated twice. Cell growth and respiration lead to the reduction of the tetrazolium dye, resulting in a blue color. The intensity of the color was proportional to the microbial growth, recorded every 15 minutes with a CCD camera, and analyzed with Omnilog software to quantify all the collected data (Wang et al., 2015).

### 2.3 Whole genome sequencing of strains T41

#### 2.3.1 Genome sequencing and assembly

The genomic DNA of *E. latusicollum* T41 strain was extracted using the SDS method, involving the disruption of fungal cells using SDS detergent to release the genomic DNA. Purification steps were performed to obtain high-quality genomic DNA for further analysis, as described by Lim et al. (2016), and its concentration was quantified using a Qubit® 2.0 Fluorometer from Thermo Scientific. Whole-genome sequencing was carried out using both the PacBio Sequel platform and Illumina NovaSeq PE150. The Illumina reads were utilized solely to enhance the accuracy of the assembly generated by PacBio reads and to reduce gaps and merge contigs, as reported by Li et al. (2023). A stringent filtering criterion was implemented to exclude low-quality reads (< 500 bp) to obtain a high-quality dataset. The preliminary assembly was performed using HGAP V. 2.3 with seed sequences selected from the long reads (≥ 6000 bp). The accuracy of the preliminary assembly was further enhanced by aligning shorter reads to the seed sequence using Blasr. The SMRT Link software’s Variant Caller module was used to correct and count variant sites in the preliminary assembly results (Li et al., 2023). Finally, the harvested DNA was subjected to agarose gel electrophoresis to confirm its integrity and purity.

#### 2.3.1 Genome annotation, gene prediction and functional annotation

The genome annotation process is a fundamental step in analyzing the genetic information of a given organism. In this study, the genome annotation process was divided into three main components, namely repeat annotation, structural annotation, and functional annotation, as outlined by Nie et al. (2023). The prediction of coding genes was accomplished using the Augustus 2.7 program, which incorporated reference gene sequences and transcript sequencing data to increase the accuracy of the predictions (Stanke et al., 2008). The identification and masking of interspersed repetitive sequences were carried out using RepeatMasker, a computational tool that aids in the recognition of transposable elements (Saha et al., 2008). Furthermore, the prediction of tandem repeats was performed using the Tandem Repeats Finder algorithm developed by Benson (1999). Transfer RNA (tRNA) genes were identified by utilizing the tRNAscan-SE program (Lowe and Eddy, 1997), while ribosomal RNA (rRNA) genes were identified using rRNAmmer (Lagesen et al., 2007). Finally, small RNA, small nuclear RNA (snRNA), and microRNA (miRNA) were predicted using BLAST searches against the Rfam database, which contains a collection of RNA families (Gardner et al., 2009; Nawrocki and Kolbe, 2009). Overall, the genome annotation process provides crucial insights into the genetic makeup of an organism, aiding in the understanding of its biology and evolution.

The functional annotation of genes was carried out using a diverse set of databases to predict their functions. These included GO (Gene Ontology) (Ashburner et al., 2000), KEGG (Kyoto Encyclopedia of Genes and Genomes) (Kanehisa et al., 2004; Kanehisa et al., 2006), KOG (Clusters of Orthologous Groups), NR (Non-Redundant Protein Database) (Li et al., 2002), TCDB (Transporter Classification Database) (Saier et al., 2014), P450 (Creˇsnar and Petriˇc, 2011), and Swiss-Prot (Amos and Rolf, 2000). A whole genome Blast search was conducted with an E-value of less than 1e-5 and a minimal alignment length percentage greater than 40% against these seven databases. Additionally, the Signal P database was used to predict secretory proteins (Petersen et al., 2011). The antiSMASH was used to analyze secondary metabolism gene clusters (Medema et al., 2011). For pathogenic fungi, pathogenicity and drug resistance analyses were performed using PHI (Urban et al., 2015) (Pathogen Host Interactions) and DFVF (Database of Fungal Virulence Factors). Furthermore, carbohydrate-active enzymes were predicted by the Carbohydrate-Active Enzymes Database (Cantarel et al., 2009).

### 2.4 Metabolic functions of phyllosphere microorganisms in tobacco affected by *Epicoccus* leaf spot

The metabolic capabilities of microbial communities in tobacco affected by *Epicoccus* leaf spot were evaluated using Biolog Eco Plates™ following the method outlined by Garau et al. (2007). The Biolog plates consisted of 96 wells, with each well containing a triplicate set of 31 diverse carbon sources, including carbohydrates, carboxylic acids, polymers, amino acids, amines, and phenolic compounds. Control wells devoid of carbon were also included (Liu et al., 2015). Sterile leaves from both healthy and diseased plants were subjected to a 0.80% saline solution, oscillated, and agitated for 2 hours at 28°C. The solution was allowed to stand for 30 minutes before use. The resulting suspensions were used to inoculate each well of the Biolog Eco plates, which were then incubated in the dark condition at 25°C for 7 days. Microbial growth was monitored by measuring the optical density (OD) at 590 nm every 24 hours using a Biolog Microstation™ reader (Biolog Inc., Hayward, CA, USA). This approach enabled the determination of catabolic profiles of the microbial communities in tobacco, providing valuable insights into their metabolic activities and potential functional roles. Analysis of the utilization of diverse carbon sources allowed for the identification of specific metabolic pathways and potential biotechnological applications of these microorganisms.

### 2.5 Community level of physiological profiles

To analyze the community-level physiological profiles (CLPP), the raw absorbance value for each well was first blanked against the control well, and negative absorbance values were set to zero, following the method proposed by Garland (1997). The blanked OD_590_ values were then used to quantify the functional diversity of microbial communities by calculating the number of wells in which the substrate was utilized by the community (with OD_590_ > 0.15), representing the richness of the community (S). This method was conducted according to the protocol established by Garau et al. (2007). To assess the overall rate of substrate utilization by microorganisms, the average well color development (AWCD) was calculated using the following equation:


(1) $$\text{AWCD}=\sum_{i=1}^{31}\left(A_{i}-A_{0}\right)/31$$

Where A_i_ is OD in each carbon source well and A_0_ is OD of the control. The growth curve of AWCD over incubation time for 168 h were performed. Then the area under the curve (AUC) of the AWCD over time was determined because according to Hackett and Griffiths (1997), the area under the color development profile summarizes the profile in a single statistic. Functional diversity assessed by the Shannon diversity index (*H*), the Simpson index (*D*) and the Pielou index (*J*) were calculated using the equation (Simpson, 1949; Pielou, 1969; Shannon, 2001; Dong et al., 2013):


(2) $$H=–\sum P_{i}\left(lnP_{i}\right)$$

(3) $$D=1-\sum P_{i}^{2}$$

(4) J=H/lns

Where P_i_ is the ratio of the relative OmniLog value of the ith carbon source to the sum of all relative OmniLog values of the whole microplate, and S is the total number of carbon sources used. All the measurements reported refer to Liu et al. (Liu et al., 2009; Liu et al., 2012).

### 2.6 Data analysis

Statistical analyses were performed using the SPSS 20 software (IBM Corp., New York, USA), a multiple comparison was performed by the LSD test (P< 0.05) to compare the diversity index of different treatments. The mean values were compared and P-value at ≦0.05 was considered to be statistically significant. Sequences obtained from each primer pair were assembled and aligned using MEGA v. 6.0 (Tamura et al., 2013) to generate consensus sequences. The alignment of these sequences, along with reference sequences downloaded from GenBank, was conducted using MAFFT v. 7 (Katoh and Standley, 2013), and manual editing was performed in MEGA v. 6.0 as needed. Maximum parsimony (MP) analyses were conducted using PAUP v 4.0 b10 (Swofford and Sullivan, 2003). Characters were assigned equal weights, and gaps were treated as missing data. An unweighted parsimony analysis was performed, utilizing the heuristic search option with TBR branch swapping and 1000 random sequence additions. The maximum number of trees (maxtrees) was set to unlimited, and branches with zero length were collapsed. All multiple parsimonious trees were saved. Clade stability was assessed through a bootstrap analysis consisting of 1000 replicates, each with 10 replicates of random stepwise addition of taxa. To determine the statistical significance of tree differences, a Shimodaira-Hasegawa test (SH test) (Shimodaira and Hasegawa, 1999) was employed. The Data Analysis 1.7 software developed by Biolog, Inc. (New York, USA) was utilized to extract optical density values obtained from OmniLog measurements. This software processed OmniLog generated data files and generated a plate file for each Phenotype MicroArray. The Microlog™ 4.01 software (Biolog Inc., Hayward, CA, USA)” to record data from the Biolog Eco Plates™. Heat maps of phenotype analysis was conducted with the software of HemI (Heatmap IIIlustrator, version 1.0) (Zhao et al., 2017). The growth curve of AWCD and diversity indices were fitted using the Origin 2021 software (OriginLab Corporation, USA).

## 3 Result

### 3.1 Growth of *E. latusicollum* strain under various culture conditions

#### 3.1.1 Characteristics colonies of *E. latusicollum* strain T41

**Figure 1:**
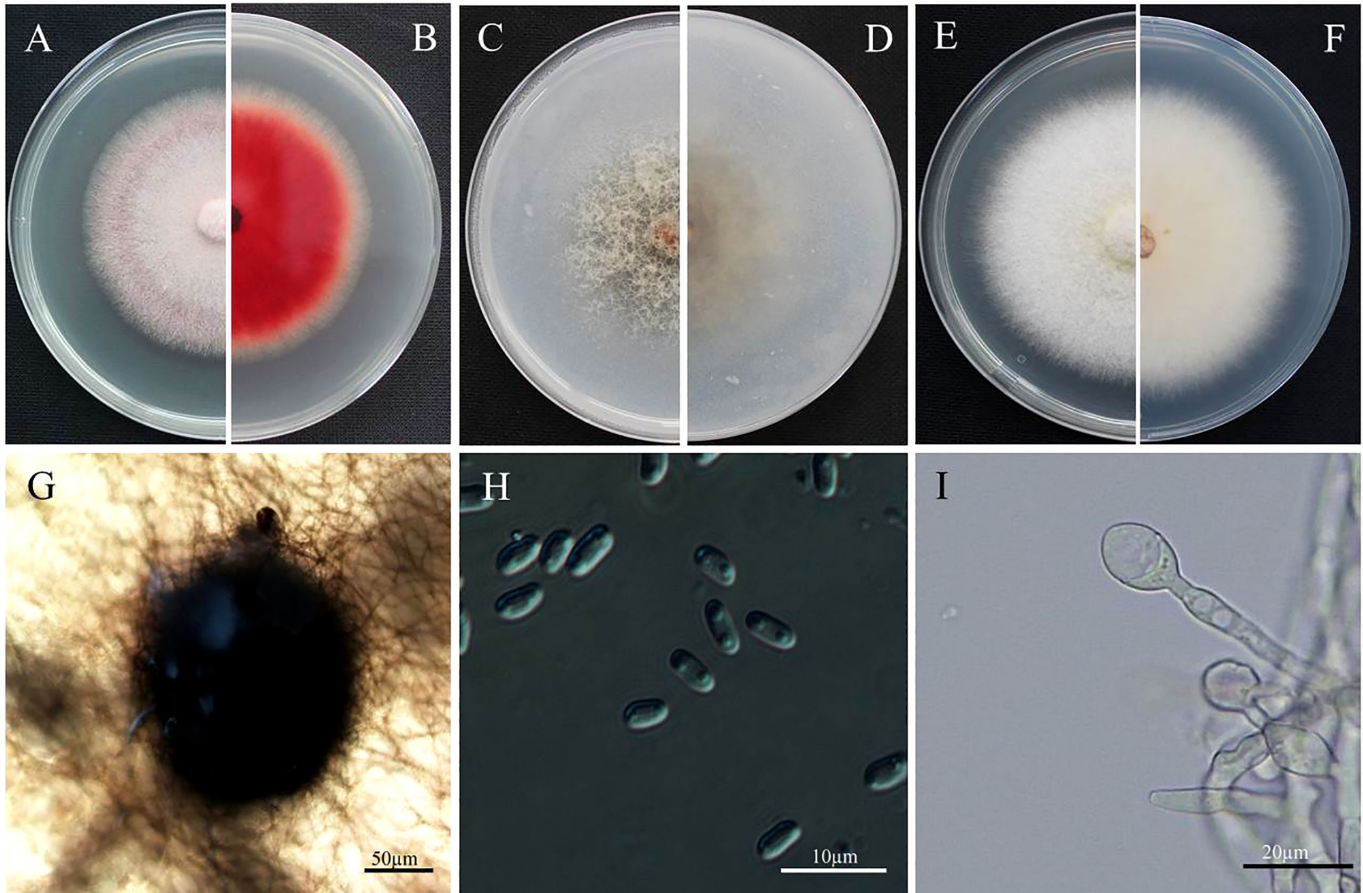
Morphological characteristics of colony and conidium of *Epicoccum latusicollum*. **(A, B)** Colony on PDA after 5 days’ incubation at 25°C in the dark (front and reverse). **(C, D)** Colony on OA after 5 days’ incubation at 25°C in the dark (front and reverse). **(E, F)** Colony on AEA after 5 days’ incubation at 25°C in the dark. **(G)** Pycnidium forming on OA. **(H)** conidia. **(I)** Swollen cells. Bars: **(G)** = 50 µm; **(H)** = 10 µm; **(I)** = 20 µm. Pycnidia: 69.2-178 µm in diameter; Conidia: 3.1-5.3 µm × 2.0-3.0 µm.

The objective of this study was to investigate the characteristics of the *E. latusicollum* strain T41, which was isolated from tobacco. The morphological features of the colonies grown on PDA medium for five days displayed a regular rounded shape with red mycelium, a white outer edge, and felted, dense, and red pigmentation on the back (**Figures 1A, B**). Similarly, on OA medium, the mycelium exhibited a white, dense, and sparse appearance at the edges, which later turned grey-brown in the middle (**Figures 1C, D**). In contrast, the mycelium on AEA medium was abundant and white, with orange-pink pigmentation observed under light exposure (**Figures 1E, F**). The growth performance of strain T41 was evaluated on the three different media types, and the findings are reported in **Table 1**. The results demonstrated that strain T41 exhibited the fastest growth rate on AEA medium, with no significant differences observed in the growth rates on OA and PDA medium (p>0.05). Moreover, spore production was observed on OA medium with alternating light and dark condition. The colonies of this isolate had woolly aerial hyphae and were white to grey eventually, producing pycnidia on OA medium. Pycnidia were black-brown, mostly spheroid, and 69.2-178 µm in diameter. Conidia were hyaline, ellipsoidal, unicellular, aseptate, and 3.1-5.3 µm × 2.0-3.0 µm (**Figures 1G–I**).

**Table 1:** Effects of different media and temperatures on the growth rate of strain T41.

Temperature/°C	PDA/mm·d^-1^	OA/mm·d^-1^	AEA/mm·d^-1^
10	3.55 ± 0.15 d^b^	3.95 ± 0.20 d^b^	5.15 ± 0.28 e^a^
15	7.21 ± 0.12 c^b^	6.10 ± 0.19 c^c^	9.90 ± 0.19 c^a^
20	9.83 ± 0.08 b^b^	8.35 ± 0.38 b^c^	11.46 ± 0.08 b^a^
25	11.08 ± 0.21 a^b^	10.85 ± 0.29 a^b^	12.21 ± 0.12 a^a^
30	0.50 ± 0.34 e^c^	3.85 ± 0.47 d^b^	6.10 ± 0.62 d^a^
35	0.00 ± 0.00 e^b^	0.19 ± 0.06 e^a^	0.06 ± 0.06 f^ab^
40	0.00 ± 0.00 e^a^	0.00 ± 0.00 e^a^	0.00 ± 0.00e^a^

A multiple comparison was performed by the LSD test (P< 0.05) to compare the diversity index of different treatments. Different lower case letters indicate statistically significant differences in the comparison of the number data between groups (p<0.05).

#### 3.1.2 Growth of *E. latusicollum* strain T41 under different temperature and light conditions

The T41 strain’s optimal temperature range for growth was determined to be between 15–30°C, with restricted growth observed above 30°C, and the optimal temperature identified as 25°C, as depicted in **Table 1**. Conversely, when the strain was subjected to adverse temperature conditions, faster growth was observed on PDA at 35°C than on OA and AEA mediums, as shown in **Table 2**. Notably, the lethal temperature for the T41 strain was determined to be 40°C, as evidenced by its inability to grow at this temperature. The impact of light on the strain’s growth was insignificant, and the pathogen did not generate spores under continuous illumination, continuous darkness, or 12 h/12 h alternating light and dark conditions after seven days of incubation in PDA medium.

**Table 2:** Optimal temperature mycelial growth rate for repositioning under adverse conditions.

Temperature/°C	PDA/mm·d^-1^	OA/mm·d^-1^	AEA/mm·d^-1^
35	12.38 ± 0.64 a^*^	9.67 ± 0.39 b^*^	10.63 ± 0.20 b^*^
40	0.00 ± 0.00	0.00 ± 0.00	0.00 ± 0.00

A multiple comparison was performed by the LSD test (P< 0.05) to compare the diversity index of different treatments. * indicates significant (P < 0.05) difference in growth rate from the optimum temperature (25°C).

#### 3.1.3 Phylogeny

Maximum likelihood phylogenetic tree analysis revealed that T41 clustered together with other *Epicoccum* isolates (**Supplementary Figure 1**), forming a distinct clade within the phylogenetic tree. This clustering pattern suggests a close evolutionary relationship among these strains and supports their classification within the genus *Epicoccum*. The bootstrap values associated with the branching patterns provided strong statistical support for the clustering of T41 with the *Epicoccum* isolates, indicating a robust phylogenetic relationship. The placement of T41 within this clade further confirms its affiliation with the *Epicoccum* genus and highlights its genetic similarity to other members of this fungal group. These findings contribute to our understanding of the evolutionary relationships and genetic diversity within the genus *Epicoccum*, shedding light on the phylogenetic positioning of T41 within this taxonomic group.

### 3.2 Characterization of strain T41 of *E. latusicollum* in terms of phenotype

A comprehensive phenotypic characterization of *E. latusicollum* isolated from tobacco was performed using PM plates 1–10, with the exception of PM 5. A total of 856 growth conditions were tested, covering a range of carbon, nitrogen, phosphorus, sulfur, osmotic, and pH environments. The isolate T41 was chosen as a representative strain based on its distinct phenotypic fingerprint. The results of the phenotypic characterization revealed that T41 was able to utilize a diverse range of carbon sources, metabolizing 61.58% of the 190 tested substrates. Specifically, the fungus was able to grow on 67 out of 95 carbon substrates on PM 1 and 50 out of 95 substrates on PM 2. In terms of nitrogen sources metabolism, T41 demonstrated remarkable versatility, utilizing 98.95% of the tested substrates (94 out of 95) on PM 3. Additionally, the fungus was capable of utilizing 98.60% of the tested nitrogen pathways, with complete utilization of all nitrogen substrates on PM 6 and PM 7, and 91 out of 95 substrates on PM 8. In terms of sulfur sources utilization, T41 was able to grow on 30 out of 35 tested substrates (85.71%) on PM 4, wells F02-H12. Similarly, the fungus demonstrated moderate utilization of phosphorus, growing on 30 out of 59 tested substrates (50.85%) on PM 4, wells A02-E12. Overall, these findings highlight the remarkable metabolic versatility of *E. latusicollum* and provide valuable insights into its ability to thrive in a wide range of environmental conditions.

#### 3.2.1 Metabolic phenotype of *E. latusicollum* strain T41 in carbon and nitrogen sources utilization

**Figure 2:**
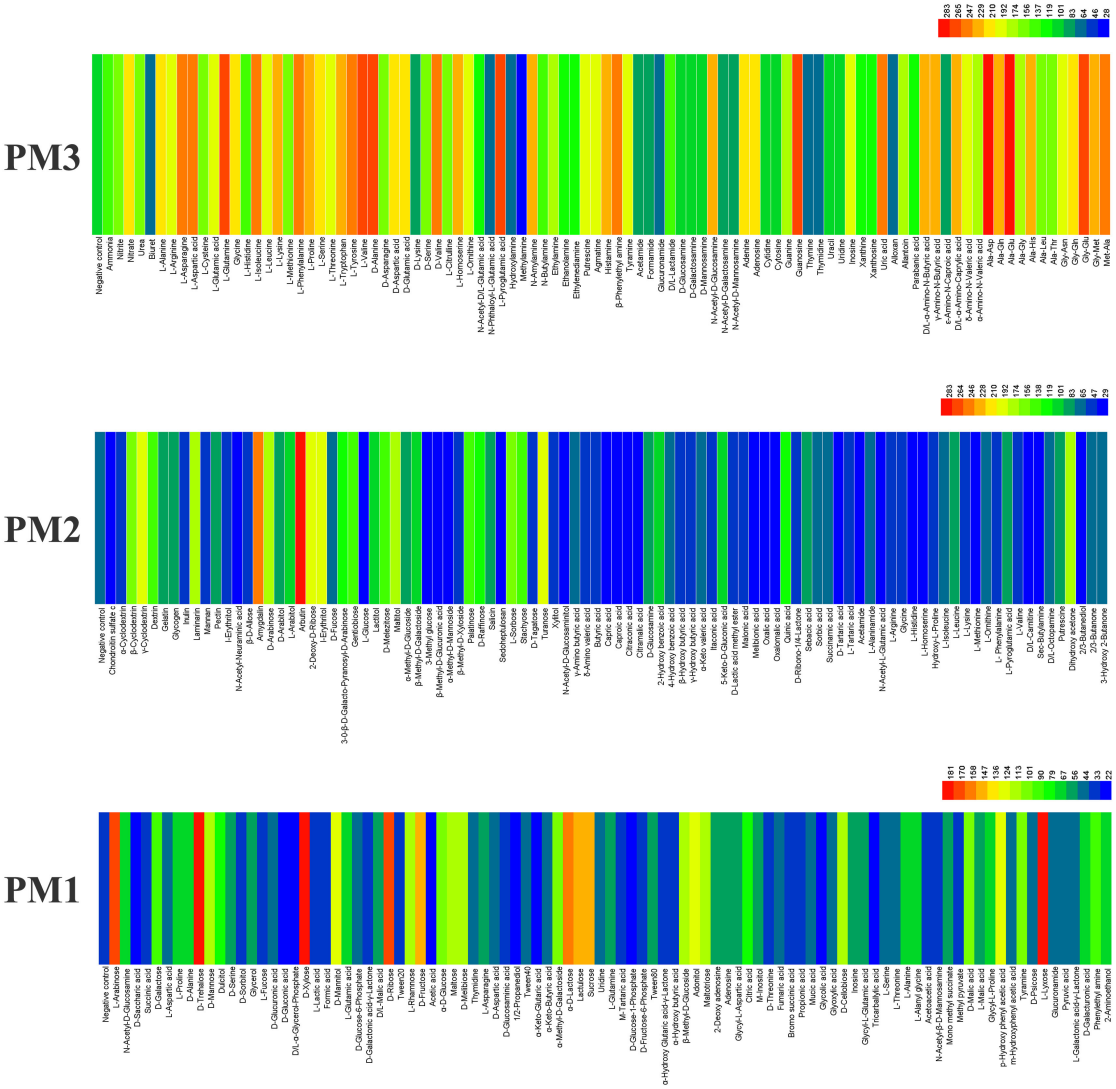
Overview of metabolic phenotypes of isolate of *Epicoccum latusicollum* on 190 carbon (C) sources and 95 nitrogen (N) sources tested. PM1, PM2 for carbon source and PM3 for its ability to metabolize 95 nitrogen substrates, respectively. Heatmap of maximum area values expressed as maximum curve area monitored during 96 h of incubation. The legend of color code from blue to green and red shades indicate low, moderate, and high utilization, respectively, assessed as arbitrary Omnilog values.

*E. latusicollum* strain T41 was found to be capable of utilizing a wide range of carbon and nitrogen sources, as shown by its ability to metabolize over 117 different carbon sources and 94 different nitrogen sources in PM plates 1–3 (**Figure 2**). The isolate was able to effectively utilize approximately 32 carbon substrates (**Supplementary Table 2**), including arbutin, amygdalin, *γ*-cyclodextrin, *I*-erythritol, 2-deoxy-*D*-ribose, *L*-lyxose, and *D*-trehalose, among others, as well as around 60 nitrogen substrates (**Supplementary Table 3**), such as Ala-Asp, Ala-Glu, *L*-glutamine, *L*-pyroglutamic acid, and *L*-valine, among others. In contrast, approximately 73 compounds could not be metabolized by the isolate, including carbon sources such as *D*-glucose-1-phosphate, *D*-saccharic acid, *D*, *L*-carnitine, and *D*, *L*-*α*-glycerol-phosphate, as well as nitrogen source methylamine.

Interestingly, the utilization rate of carbon sources was lower than that of nitrogen sources, suggesting that *E. latusicollum* may have evolved to rely more heavily on nitrogen-based metabolism. This finding is consistent with previous studies on fungal metabolism, which have shown that fungi often prioritize the utilization of nitrogen over carbon sources. Overall, these results provide valuable insights into the metabolic versatility of *E. latusicollum* and highlight its ability to utilize a diverse range of carbon and nitrogen sources, which may play a key role in its adaptation to a variety of ecological niches.

#### 3.2.2 Metabolic phenotype of *E. latusicollum* strain T41 in peptide nitrogen sources utilization

**Figure 3:**
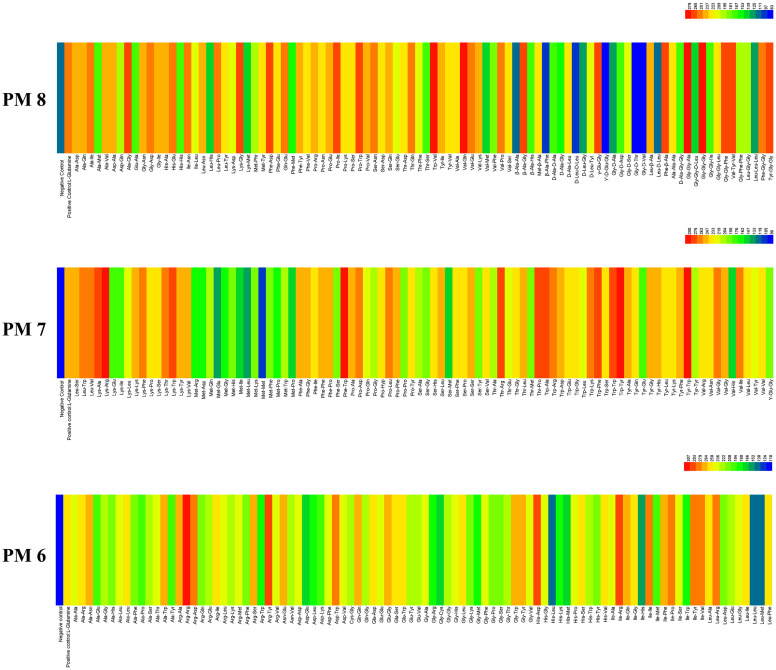
Overview of metabolic phenotypes of isolate of *Epicoccum latusicollum* on 285 nitrogen pathways tested. PM6, PM7 and PM8 for nitrogen pathways. Heatmap of maximum area values expressed as maximum curve area monitored during 96 h of incubation. The legend of color code from blue to green and red shades indicate low, moderate, and high utilization, respectively, assessed as arbitrary Omnilog values.

*E. Latusicollum* strain T41 showed remarkable metabolic flexibility with respect to its utilization of different peptide nitrogen sources, as evidenced by the results from PM plates 6 to 8. The pathogen was found to be capable of utilizing 285 different peptide nitrogen sources, which indicates that it is able to grow on a diverse range of amino acid combinations. Of these peptide nitrogen sources, 281 were able to support the growth of the pathogen (**Figure 3**), with both PM6 and PM7 able to be metabolized totally. Interestingly, more than 214 efficient nitrogen pathways were identified, including Arg-Arg, His-Asp, Arg-Tyr, Trp-Tyr, Phe-Trp, Tyr-Trp, Gly-Gly-Ala, Val-Gln, and Trp-Val, among others (**Supplementary Table 4**). These results suggest that *E. latusicollum* has evolved sophisticated mechanisms for scavenging and utilizing a diverse range of nitrogen sources, which may allow it to thrive in various ecological niches. In comparison to carbon and other sources, the utilization rate of peptide nitrogen sources was found to be particularly high. Only four peptide nitrogen sources significantly inhibited the growth of the pathogen, indicating that nitrogen pathways may be a preferred source of nitrogen for *E. latusicollum*.

#### 3.2.3 Metabolic phenotype of *E. latusicollum* strain T41 in phosphorus and sulfur sources utilization

**Figure 4:**
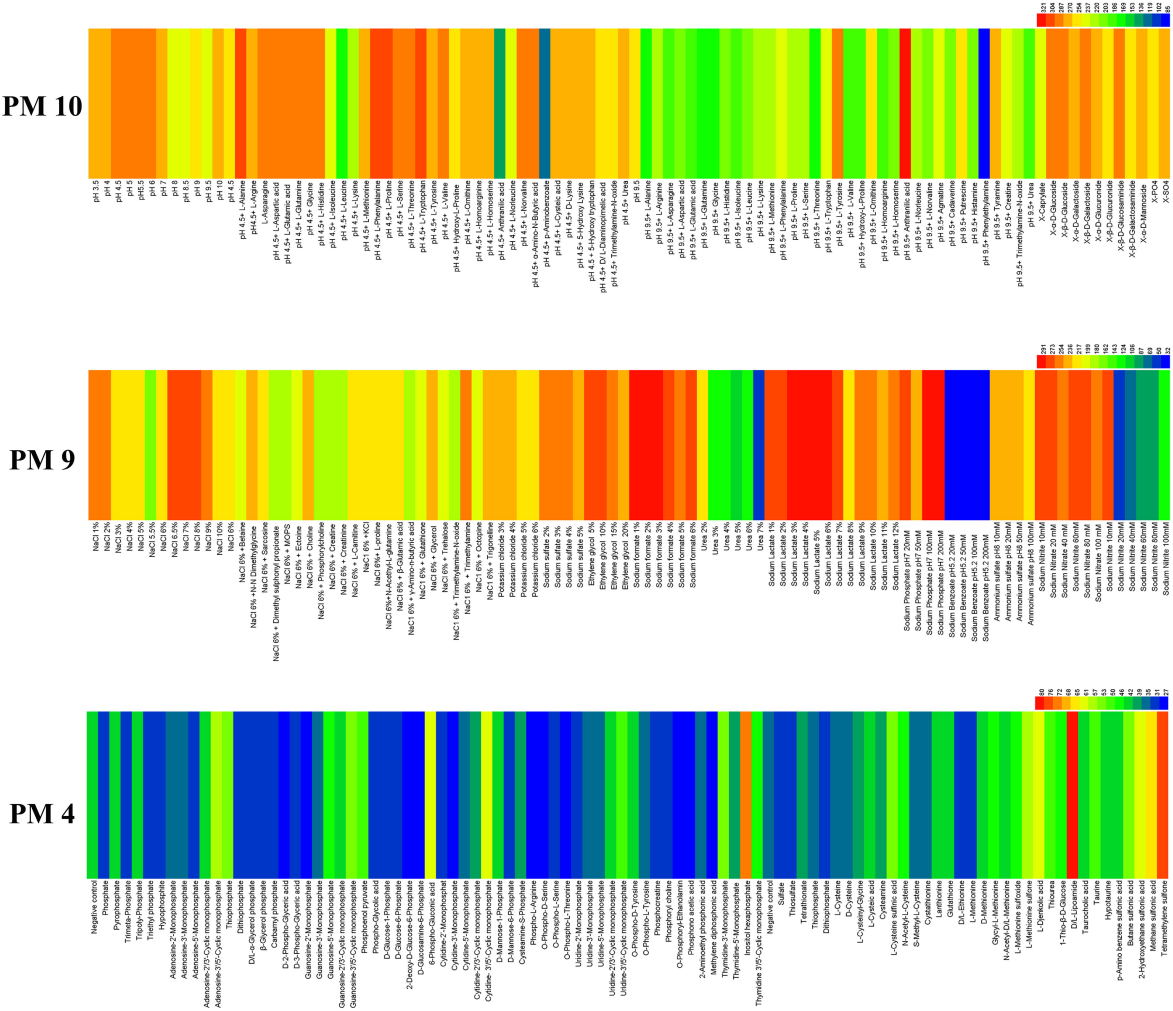
Overview of metabolic phenotypes of isolate of *Epicoccum latusicollum* on 59 phosphorus substrates, 35 sulfur substrates, 96 osmotic, ionic conditions, and 96 pH environments tested. PM4 for its ability to metabolize 59 phosphorus compounds and on 35 different sulfur substrates, PM9 and PM10 for its the fungal growth under various stress conditions was tested, respectively. Heatmap of maximum area values expressed as maximum curve area monitored during 96 h of incubation. The legend of color code from blue to green and red shades indicate low, moderate, and high utilization, respectively, assessed as arbitrary Omnilog values.

The ability of *E. latusicollum* strain T41 to metabolize phosphorus compounds and sulfur substrates was assessed using the PM 4 plate (**Figure 4**). The results showed that the tested isolate was more efficient in utilizing sulfur substrates than phosphorus compounds. Out of the phosphorus compounds tested, only four were effectively metabolized, including inositol hexaphosphate, 6-phospho-gluconic acid, cytidine-3’,5’-cyclic monophosphate, and adenosine-3’,5’-cyclic monophosphate. On the other hand, more than seven sulfur substrates were effectively utilized, including *D*, *L*-lipoamide, tetramethylene sulfone, methane sulfonic acid, *L*-djenkolic acid, 2-hydroxyethane sulfonic acid, and *L*-methionine sulfone (**Supplementary Table 5**). In contrast, five sulfur substrates were not utilized, including *D*, *L*-ethionine, *L*-methionine, sulfate, dithiophosphate, and thiosulfate. Overall, the results suggest that *E. latusicollum* has a higher capacity to utilize sulfur substrates than phosphorus compounds.

#### 3.2.4 Metabolic phenotype of *E. latusicollum* strain T41 in osmotic/ion and pH effects

The adaptability of T41, a fungal isolate, was assessed using PM 9 and PM 10 plates under diverse stress conditions (**Figure 4**). The results demonstrated the wide range of metabolic capabilities exhibited by *E. latusicollum*, enabling its growth in the presence of different osmolytes. Notably, the pathogen exhibited optimal growth in the presence of sodium lactate, with an optimal concentration of 5% and a range of 1% to 12%. Furthermore, it effectively utilized sodium formate, sodium phosphate at pH 7, NaCl, and sodium nitrate, with optimal concentrations ranging from 1% to 20 mM (**Supplementary Table 6**). Moreover, *E. latusicollum* demonstrated efficient metabolism of various compounds, including trimethylamine, *N*-*N* dimethylglycine, and choline, even in the presence of NaCl at 6% concentration.

The pH range where *E. latusicollum* grew most effectively was 3.5 to 10.0, with an optimal pH of approximately 6.0. When tested with different amino acids under the stress of pH 4.5, the pathogen grew effectively in most tests (plate PM 10, wells B1 to B12, C1 to C12, and D1 to D12), whereas no significant growth was observed under the stress of pH 9.5, except when combined with anthranilic acid (plate PM 10, wells E1 to E12, F1 to F12, and F1 to F12) (**Supplementary Table 6**). The decarboxylase and deaminase activities of *E. latusicollum* were also evaluated on PM 10 in wells of B1-D12 and E1-G12, respectively, under the stress of different amino acids at pH 4.5 and 9.5. The pathogen exhibited active decarboxylase activity but poor deaminase activity.

### 3.3 Whole genome sequencing and statistical analysis

After meeting quality control criteria, a total of 865,706 clean reads were obtained for the *E. latusicollum* isolate T41, with an N50 read length value of 10,563 bp, generating ~ 213.61 average coverage and 7,174,496,045 clean bp for subsequent analyses. A PacBio RS library was also prepared, generating 73,744 total PacBio reads and 1,162,320,095 total bp after filtering, with the largest length being 3,347,270 bp and N50 and N90 lengths of 1,588,440 and 667,065 bp, respectively. Gene length statistics were presented in **Supplementary Figure 2**. The genome sequence of strain T41 was polished and assembled into 23 contigs, with an N50 length of 1,982,256 bp, maximum contig length of 4,035,028 bp, and a total contig length of 33,577,935 bp. After quality control, the number of contigs was reduced to 22, with an N50 length of 1,982,625 bp, maximum contig length of 4,035,947 bp, and total length of 33,587,228 bp. The G+C content was 52.00% based on read sequence data being processed with the condition of 15-mers using the K-mer statistical method (**Table 3**). The whole-genome sequence and annotation of *E. latusicollum* isolate T41 have been deposited at NCBI (https://www.ncbi.nlm.nih.gov/) with accession PRJNA645278; BioSample SAMN15501750.

**Table 3:** Genome assembly statistics of *Epicoccum latusicollum* isolate T41.

Assembly parameters	T41
Sequencing platform	PacBio and Illumina PE150
Assembly method	HGAP V. 2. 3
Genome size (Mb)	33.59
Sequencing coverage	213.6 x
Number of contigs	22
Average contig length (bp)	1,526,692
Contig N50 (bp)	1,982,625
Maximum contig length (bp)	4,035,947
Number of all Contigs	23
Maxium Contig length (bp)	4,035,028
Contig N50 length (bp)	1,982,256
G+C content (%)	52.00

#### 3.3.1 Genome component analysis of coding genes, repeat sequence and ncRNA

The genome assembly of the organism was annotated, resulting in the identification of 10,821 genes. The total length of these genes was 16,688,112 bp, and the average length was 1,542 bp, accounting for almost half (49.69%) of the genome. Transposable elements (TEs) were also identified in the genome assembly, representing 1.23% of the total genome with a total length of 412,616 bp. A total of 1679 TE families were analyzed with RepeatMasker, of which 99% (1663) belonged to the known TEs. These included 1287 retrotransposons (Class I) and 376 DNA transposons (Class II), which could be further divided into different groups and subgroups, such as LINE, LTR, SINE, etc. Retrotransposons, in particular, were found to be abundant in fungi, and they might play a role in genome expansion due to their redundancy and tolerance for mutations (**Table 4**).

**Table 4:** Genomic component statistics of *Epicoccum latusicollum* isolate T41.

Gene prediction				
Gene number	10,821			
Gene total length (bp)	16,688,112			
Gene average length (bp)	1,542			
Gene/genome (%)	49.69			
Repeat				
Type	Number	Total Length(bp)	In Genome(%)	Average length(bp)
LTR	872	252,576	0.752	294
DNA	376	128,679	0.3831	345
LINE	349	31,743	0.0945	105
SINE	66	4,102	0.0122	62
Unknown	17	1,318	0.0039	78
RC	16	1,809	0.0054	113
Total	1,696	413,934	1.2324	253
Type	Number	Repeat Size(bp)	Total Length(bp)	In Genome(%)
**TR**	3,049	1~1,643	169,232	0.5039
Minisatellite DNA	2,203	10~60	106,231	0.3163
Microsatellite DNA	445	2~6	20,858	0.0621
ncRNA	Type	Number	Average length(bp)	Total Length(bp)
	tRNA	160	89	14,345
rRNA	5s(denovo)	51	116	5,917
	5.8s(denovo)	0	0	0
	18s(denovo)	0	0	0
	28s(denovo)	1	3,532	3,532
	sRNA	2	236	472
	snRNA	20	140	2,816
	miRNA	0	0	0

**Figure 5:**
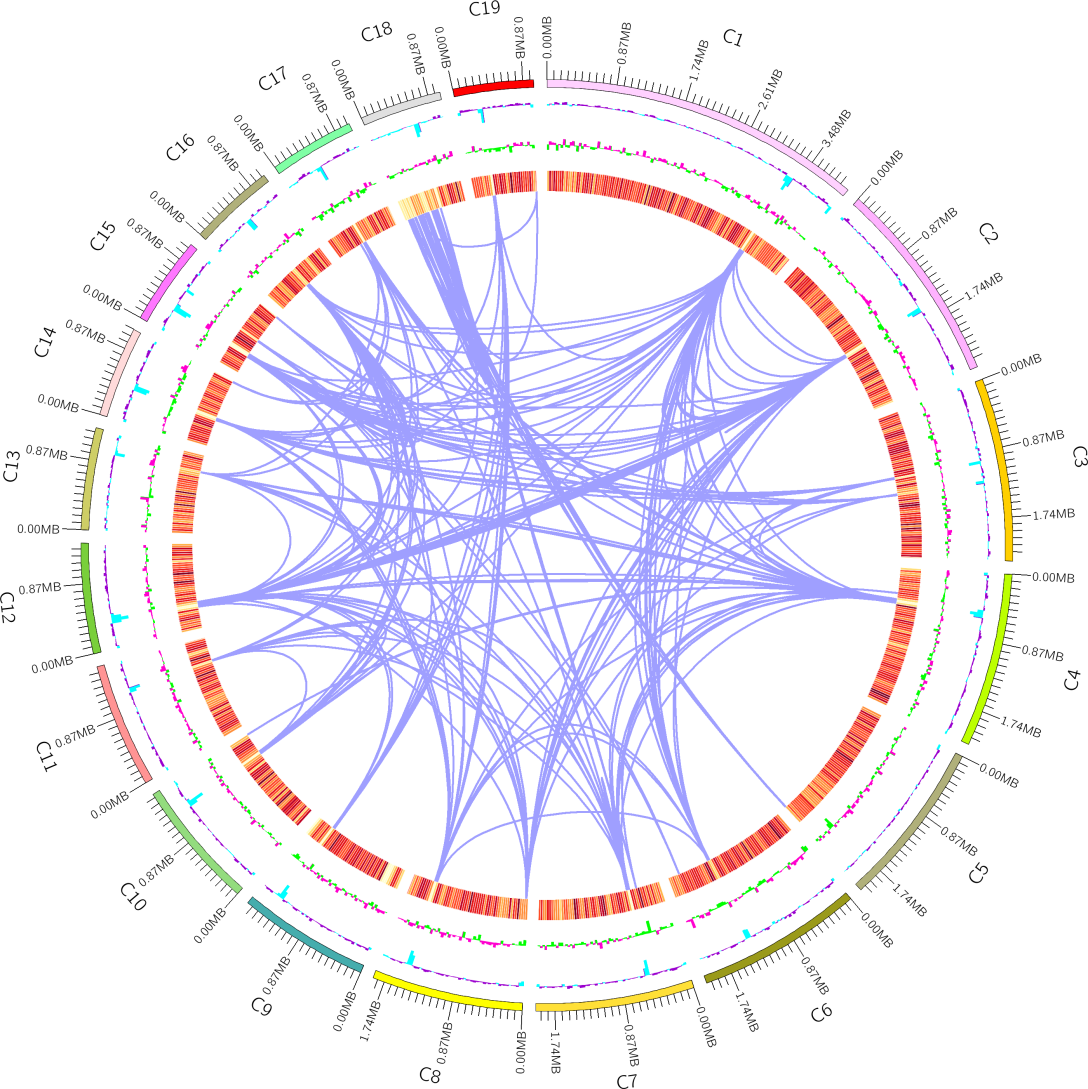
Genome Mapping. First circle: the outermost circle is the genome sequence position coordinates; Second circle: genomic GC content: the GC content is counted with a window of 200000 bp and a step of (200000) bp. The inward l blue part indicates that the GC content of this region is lower than the average GC content of the whole genome, the outward purple part is the opposite, and the higher the peak indicates a greater difference from the average GC content; Third circle: genomic GC skew value: window (200000) bp, step (200000) bp, the specific algorithm is G-C/G+C, the inward green part indicates that the content of G in the region is lower than the content of C, the outward pink part is the opposite; The fourth - seventh circle: Gene density (with a window of 200000 bp and a step length of 200000bp to count the gene density of coding genes, rRNA, snRNA, tRNA respectively, the darker the color, the greater the density of genes within the window); Innermost circle: donors and acceptors of segmental duplications on fungus chromosomes are connected by purple lines.

Furthermore, non-coding RNAs (ncRNA) were identified in the genome assembly, with a total of 234 ncRNAs representing only 0.2% of the genome size (**Table 4**). These molecules resemble mRNAs in structure and function, but they do not encode proteins. Instead, they regulate the transcription and translation of mRNAs in close proximity to them. In addition, the genome mapping (**Figure 5**) provided a visual representation of the whole genome structure of the organism, including the assembled genome sequence, predicted coding genes, and other known data patterns. Overall, the annotation and analysis of the genome assembly provided valuable insights into the genetic makeup of the organism and shed light on various genomic features, such as TEs, TR units, and ncRNAs.

#### 3.3.2 Gene function analysis

The majority of genes (92.74%) were annotated using the databases described in the methods section. This paper focuses on genes involved in metabolic processes. Approximately 86.27% (9335) of all predicted genes were annotated by the KEGG pathway, and among these genes, those involved in metabolism accounted for a major proportion of 27.93% (2607) of the total predicted genes (**Supplementary Figure 3**). Genes classified into functional categories according to KOG analysis accounted for 19.41% (2100), and among these genes, those involved in metabolic processes accounted for 5.45% (590) of the total predicted genes, and about 0.43% (46) of the predicted genes were related to the biosynthesis, transport and catabolism of secondary metabolites (**Supplementary Figure 4**). The proportion of genes encoding CAZymes was 5.11% (553); these genes contribute to the process of substrate degradation in the nutrition of fungal development and reproduction. Among the genes associated with CAZymes, 276 genes encoding glycoside hydrolases (GH) accounted for the largest proportion of the total number of predicted genes (2.55%), followed by 102 genes encoding glycosyltransferases (GT) (0.94%) and then 66 genes encoding glycosyltransferases (GT) (0.72%). Genes that acted as auxiliary activities (AA) accounted for 0.82%. In addition, genes belonging to carbohydrate esterases (CEs) and polysaccharide lyases (PLs) accounted for a much lower percentage of the total number of predicted genes, 0.41% and 0.14%, respectively (**Supplementary Figure 5**). Overall, the study provides valuable insights into the genetic and molecular mechanisms underlying fruiting body development. The identification of genes involved in metabolic processes and CAZymes genes will be useful for further research in this area. These findings could help to better understand the metabolic processes involved in fruiting body development, and how CAZymes genes play a role in this process. Further studies can explore the potential applications of these genes in biotechnology and agriculture.

### 3.4 Assessment of the pathogenicity of *E. latusicollum* and its impact on the metabolic functions of tobacco phyllosphere microorganisms

**Figure 6:**
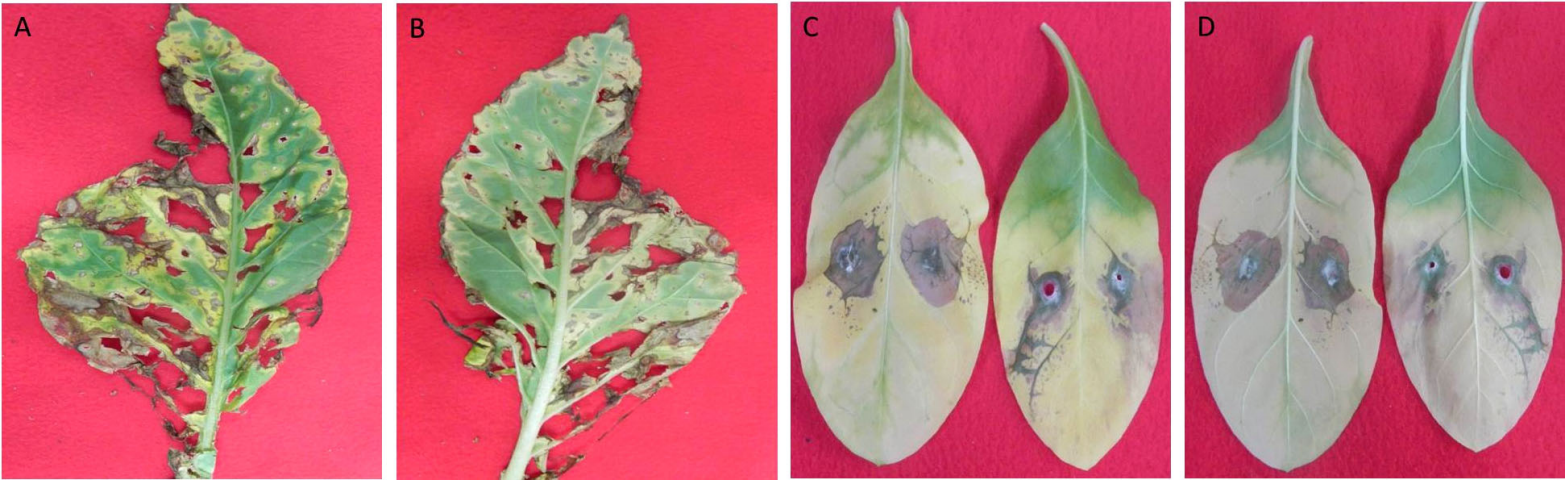
Observation and inoculation of tobacco leaf spot symptoms induced by *E latusicollum.* Symptoms of tobacco leaf spot, induced by the pathogen *E. latusicollum*, were observed on both the upper **(A)** and lower **(B)** surfaces of the leaves. Inoculation of the leaves with mycelial plugs occurred following a 7-day incubation period at 28°C, resulting in visible symptoms on the upper **(C)** and lower **(D)** leaf surfaces.

To evaluate the pathogenicity of T41, detached tobacco leaves were employed as a model. The results demonstrated that T41 induced visible symptoms on the leaves. The observed symptoms included sandy beige lesions that were elliptical or irregular in shape, characterized by a brown edge and surrounded by yellow halos. These symptoms exhibited a progressive pattern, spreading from the inoculation sites to the adjacent areas of the leaves (**Figure 6**). In order to comprehensively investigate the impact of *E. latusicollum* strain T41 on the metabolic function of phyllosphere microorganisms, detailed results were presented in **Supplementary Table 7**. The table depicted the effect of *E. latusicollum* on the metabolism of tobacco phyllosphere microorganisms in both healthy and diseased leaves. Furthermore, **Table 5** provided an overview of carbon substrates in the Biolog ECO microplates, categorized by their biochemical properties. The analysis of the data showed that in healthy T41 samples, all carbon sources were metabolized, except for 4-hydroxy benzoic acid and *α*-ketobutyric acid. Certain carbon sources such as *D*-xylose, 2-hydroxy benzoic acid, *L*-phenylalanine, *L*-threonine, and putrescine were found to be inefficiently metabolized. Interestingly, in diseased T41 samples, 29 carbon sources were unable to be metabolized, with only tween 40 exhibiting some degree of metabolizing ability. These findings suggested a significant suppression of the metabolic ability of carbon sources in phyllosphere microorganisms upon tobacco infection by *E. latusicollum*. Further research is required to elucidate the potential changes in the interleaf microbial communities on the surface, which may underlie the observed inhibition.

**Table 5:** Carbon substrates in Biolog ECO microplates.

Polymers	
*α*-Cyclodextrin	*γ*-Hydroxybutyric Acid
Glycogen	*α*-Ketobutyric Acid
Tween 40	Itaconic Acid
Tween 80	*D*-Malic Acid
**Carbohydrates**	Pyruvic Acid Methyl Ester
*D*-Cellobiose	**Amino acids**
*I*-Erythritol	*L*-Arginine
*D*-Galactonic Acid *γ*-Lactone	*L*-Asparagine
*N*-Acetyl-*D*-Glucosamine	Glycyl-*L*-Glutamic Acid
Glucose-1-Phosphate	*L*-Phenylalanine
*β*-Methyl-*D*-Glucoside	*L*-Serine
*D*,*L*-*α*-Glycerol phosphate	*L*-Threonine
*α*-*D*-Lactose	**Amine**
*D*-Mannitol	Phenyl ethylamine
*D*-Xylose	Putrescine
*D*-Galacturonic Acid	**Phenolic compounds**
*D*-Glucosaminic Acid	2-Hydroxy Benzoic Acid
**Carboxylic acids**	4-Hydroxy Benzoic Acid

Assignment to biochemical categories follows that of Insam (1997).

The Biolog plates consisted of 96 wells, with each well containing a triplicate set of 31 diverse carbon sources, divided into six categories, including carbohydrates, carboxylic acids, polymers, amino acids, amines, and phenolic compounds.

### 3.5 Diversity index of microbial metabolic functions in tobacco *Epicoccus* leaf spot

**Figure 7:**
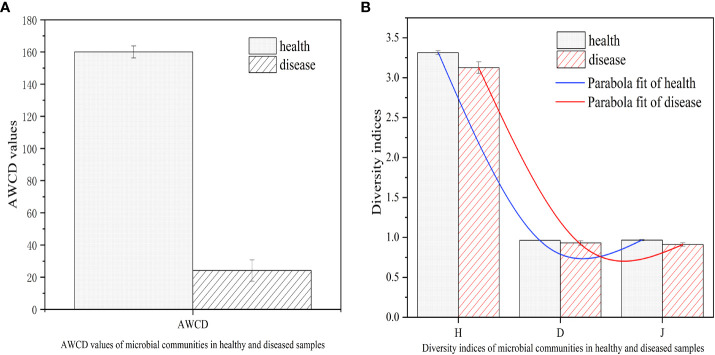
AWCD values and diversity indices of microbial communities in healthy and diseased samples. **(A)** AWCD values of microbial communities in healthy and diseased samples. **(B)** Diversity indices of microbial communities in healthy and diseased samples. Average well color development (AWCD); Shannon-Wiener indexes (H); Simpson indexes (D); Pielou indexes (J).

The color change rate of microorganisms from healthy and infected tobacco *Epicoccus* leaf spot samples was measured using the OmniLog system. As depicted in **Figure 7**, the overall trend of the average color change rate of each group showed a significant difference between infected and healthy samples. The average well color development (AWCD) of microorganisms in the infected tobacco leaf spot was much lower compared to that of the healthy samples. This observation could be indicative of the compromised metabolic activity of microorganisms in the tobacco *Epicoccus* leaf spot, which could be attributed to the presence of *E. latusicollum*, the causative agent of the disease.

To further evaluate the microbial community structure of the infected and healthy tobacco leaves, the Shannon-Wiener (*H*), Simpson (*D*), and Pielou (*J*) indices were computed. The results showed that the microbial diversity and evenness of the *Epicoccus* leaf spot were comparable to those of the healthy samples, although the values were slightly higher in the healthy samples. This suggests that although the microbial community structure of the *Epicoccus* leaf spot was not significantly different from that of the healthy samples, there could be subtle changes in the community composition and function due to the presence of the pathogenic fungus. These findings highlight the importance of assessing the ecological and functional impacts of plant-pathogen interactions on the phyllosphere microbial communities, which could have implications for plant health and disease management strategies.

## 4 Discussion

*Epicoccum latusicollum* is a newly discovered fungus (Chen et al., 2017) that causes a severe foliar disease (Guo et al., 2021). Notwithstanding the considerable impact of this fungus, there is limited research on *E. latusicollum* in China and other countries, with only a few taxonomic studies and scattered literature references available (Han et al., 2019). The primary objective of the present investigation is to conduct an extensive and systematic evaluation of the growth conditions, metabolic phenomics, and genomic characteristics of *E. latusicollum*, additionally, this study aims to elucidate the metabolic functions of phyllosphere microorganisms in tobacco affected by *Epicoccus* leaf spot and their modulation in response to the disease.

The fungus *E. latusicollum*, which is recognized as a causative agent of tobacco *Epicoccus* leaf spot, displays a broad spectrum of temperature tolerance for growth, albeit being more susceptible to high temperatures. Prior studies have suggested that *Epicoccum* strains exhibit intolerance to high temperatures and may encounter mortality at approximately 40°C (Huang et al., 2021a; He et al., 2022). Specifically, we observed that the *E. latusicollum* strain’s growth was notably sluggish at temperatures of 30°C and above, yet the strain could endure these conditions and eventually return to normal growth at temperatures of 25°C or below. Although the effects of various growth media on growth rate have been extensively studied in prior research (He et al., 2022), less attention has been paid to the influence of different media on conidial production. In our preliminary exploration, we investigated the effects of different media on conidia production and discovered that PDA medium did not stimulate the production of conidia, whereas OA medium did. However, further investigations are warranted to elucidate the impacts of diverse growth conditions on conidia production across various media types. The studies of *Epicoccum* strains revealed a commonality in the metabolism of the strains in acidic environments, as shown by both traditional methods (Huang et al., 2021a; He et al., 2022) and the Biolog Phenotype MicroArray technique, faster metabolism and growth rate under acidic conditions. In the present study, strain T41 exhibited a wide range of adaptability and active metabolism across environments with pH values ranging from 3.5 to 9.5, as demonstrated by plate PM 9. Notably, deaminase and decarboxylase activities were determined at pH values of 4.5 and 9.5, respectively, under different amino acid stresses, with decarboxylase activity exceeding deaminase activity. These findings are in agreement with those reported for Tobacco brown spot (Liu et al., 2022). By utilizing Biolog Phenotype MicroArray plates PM 1-4 and PM 6-10, we identified almost 857 phenotypic traits for *E. latusicollum* strain T41. Our results revealed that the strain was capable of thriving in a wide range of environments, with PM 1/PM 2, PM 3, PM 9, and PM 10 being the plates that were significantly metabolized, these findings were consistent with those of other researchers who have studied microorganisms, such as Friedl et al. (2008) and Wang et al. (2018). However, compared to *Rhizopus oryzae* (Li et al., 2023) and *Phytophthora parasitica* (Wang et al., 2015), *E. latusicollum* showed lower carbon source utilization. This suggested that the PM technique allowed for the examination of various factors related to how environmental stressors impact pathogen activity, making it possible to assess whether it could be useful for disease management in agriculture. Reducing the availability of carbon sources utilized by *E. latusicollum* or increasing the amount of sources that cannot be metabolized by the pathogen in the field may decrease the damage caused by *Epicoccus* leaf spot. *E. latusicollum* exhibited higher utilization of nitrogen sources when compared to *Botrytis cinerea* (Wang et al., 2018), indicating that the strain had evolved sophisticated mechanisms for scavenging and utilizing a diverse range of nitrogen sources, allowing it to thrive in various ecological niches.

The present study involves a genomic analysis of *E. latusicollum*, a fungal pathogen known to cause economic crop damage, particularly in tobacco. However, recent research suggests that *E. latusicollum* isolated from *R. roxburghii* may possess potent antimicrobial properties and could prove beneficial, exhibited antimicrobial activity against several plant pathogens, including *Lasiodiplodia theobromae*, *Botryosphaeria dothidea*, *Colletotrichum capsici*, *Pyricularia oryzae*, *Rhizoctonia solani*, and *Fusarium oxysporum* (Zhang et al., 2022). The genome size of the T41 strain aligns with previous estimates for *Epicoccum*, which range from 33-35 Mbp (Fokin et al., 2017; Oliveira et al., 2017; Zhang et al., 2022). The GC content of both the T41 and HGUP191049 strains were comparable, indicating a degree of relatedness between the strains. Both strains were sequenced using a combination of second-generation Illumina and third-generation PacBio sequencing technology, and the whole-genome sequencing data showed similarities in terms of gene size, protein-coding genes, and number of assemblies, although the assembly levels differed in terms of contigs and scaffolds.

Analysis of the ‘increased pathogenicity (hypervirulence)’ genes, which are essential pathogenic factors according to the Pathogen Host Interactions (PHI) Database, revealed differences in the number of such genes between different nutrient modes of the same *Epicoccum* species, such as *E. latusicollum* (T41 and HGUP191049). Furthermore, it was found that endophytic strains of the same species may contain more ‘loss of pathogenicity phenotypic genes’ than a pathogen. In the case of *E. latusicollum*, the endophytic HGUP191049 strain had more of these genes than the pathogenic T41 strain, which may contribute to differences in their pathogenicity within the species (Zhang et al., 2022). **Supplementary Figure 6** provides detailed information on this topic.

The utilization ability of microbial communities inhabiting the phyllosphere microenvironment of tobacco leaves was investigated using substrate utilization profiling (BIOLOG) by the researchers. The results of the BIOLOG assay demonstrated that the microbial communities had a robust ability to utilize carbohydrates, followed by polymers, carboxylic acids, amino acids, and amines, with minimal utilization of phenolic compounds. Specifically, *D*-cellobiose, *D*-mannitol, *N*-acetyl-*D*-glucosamine, glucose-1-phosphate, and *D*-galactonic acid *γ*-lactone were the most utilized carbohydrates in the healthy samples. The finding of Dai et al. (2022) corroborated this result, as their investigation of microbial communities in Tobacco brown spot revealed similar carbohydrate utilization patterns and weak phenolic compound (4-hydroxy benzoic acid) utilization. Notably, a comparison of healthy and diseased samples demonstrated that the healthy group had higher rates of carbon source utilization than the diseased group. This finding may indicate compromised metabolic activity of microorganisms in the tobacco leaf spot, attributable to the presence of the pathogen, the causative agent of the disease. However, the utilization ability of polymers was stronger in the microbial communities inhabiting tobacco *Epicoccus* leaf spot than those in Tobacco brown spot (Dai et al., 2022). To further assess microbial community diversity, the study computed diversity indices. The results showed that the microbial diversity and richness of the *Epicoccus* leaf spot were comparable to those of the healthy group, albeit slightly higher in the healthy samples. These results suggested that although the microbial community structure of the *Epicoccus* leaf spot was not significantly different from that of the healthy group, subtle changes in community composition and function could occur due to the presence of the pathogenic fungus.

The data obtained in this research on *E. latusicollum* and its metabolic versatility, optimal growth conditions, and genomic information can play a vital role in developing integrated strategies for disease control and breeding for disease resistance of tobacco *Epicoccus* leaf spot. Understanding the optimal carbon, nitrogen, phosphorus, and sulfur sources for the growth of *E. latusicollum* allows for targeted manipulation of these factors to limit the fungus’s growth and spread. By restricting the availability of specific carbon sources like arbutin and amygdalin, and nitrogen sources such as Ala-Asp and Ala-Glu, through modified agricultural practices or nutrient management strategies, the growth and proliferation of *E. latusicollum* can be effectively impeded. Additionally, adjusting the composition or application of phosphorus and sulfur fertilizers based on the fungus’s optimal requirements can create nutrient imbalances that hinder its growth and reduce infection potential. Manipulating the availability of optimal carbon, nitrogen, phosphorus, and sulfur sources creates an unfavorable environment for *E. latusicollum*, supplementing other preventive measures like crop rotation, sanitation practices, and the use of resistant cultivars. This targeted approach to nutrient management is crucial for integrated disease control strategies. The genomic information obtained from sequencing *E. latusicollum* strain T41 provides valuable insights into the genetic basis of pathogenicity and potential mechanisms of resistance. By identifying genes associated with virulence, pathogenicity, or resistance, researchers can further investigate these factors for potential utilization in breeding programs or genetic manipulation. This knowledge contributes to the development of resistant tobacco cultivars, enhancing long-term disease management and reducing crop losses. Furthermore, the study’s findings on the metabolic functions of phyllosphere microorganisms in diseased tobacco leaves offer insights into interactions between *E. latusicollum* and other microorganisms. Understanding the metabolic capabilities of these microorganisms can aid in the identification of potential biocontrol agents or the development of probiotic treatments. Exploiting microorganisms that efficiently metabolize carbon sources that *E. latusicollum* struggles to utilize can limit its resources and inhibit its growth. By integrating the research findings into practical strategies such as cultural practices, genetic manipulation, and biocontrol approaches, new and effective disease control and management strategies can be developed. These strategies have the potential to reduce the incidence and severity of tobacco *Epicoccus* leaf spot, minimize yield losses, and enhance overall crop productivity and quality.

## 5 Conclusion

Tobacco *Epicoccus* leaf spot, a notorious foliar disease instigated by *Epicoccum latusicollum*, has been reported to cause significant losses in tobacco quality and yield in the southwest region of China. In this study, we aimed to investigate the physiological and genomic characteristics of the aforementioned pathogen. Specifically, we identified the optimal culture conditions for *E. latusicollum* isolate T41, including the alkyl ester agar medium for mycelial growth and oatmeal agar medium for sporulation, with the optimal temperature for mycelial growth being 25°C and the lethal temperature being 40°C. Moreover, we analyzed the metabolic phenotypes of *E. latusicollum* using Biolog Phenotype MicroArray and observed the utilization of the majority of the tested carbon sources, with arbutin and amygdalin being the preferred carbon sources. Ala-Asp and Ala-Glu were identified as the most suitable nitrogen sources among the tested nitrogen sources. Remarkably, *E. latusicollum* exhibited remarkable adaptability, metabolizing 61.46% of carbon sources, 99.48% of nitrogen sources, 85.71% of sulfur sources, and 50.85% of phosphorus sources, as well as displaying an active metabolism in a wide range of environments, including those with varying pH values and different osmolytes. Additionally, the genome of T41 was sequenced using Illumina HiSeq and Pacific Biosciences technologies and assembled into 33.59 Mbp with a N50 value of 1.58 Mbp, in which a total of 10,821 genes were predicted and analyzed using various databases. We further examined the metabolic functions of phyllosphere microorganisms in diseased tobacco leaves caused by *E. latusicollum* using Biolog Eco microplate and identified a significant incapacity to efficiently metabolize 29 carbon sources, except for tween 40. This study provides novel insights into the structural and functional characteristics of phyllosphere microbiota and underscores crucial challenges for future research, as well as a theoretical foundation for the integrated control and breeding for disease resistance of tobacco *Epicoccus* leaf spot.

## Data availability statement

The data presented in the study are deposited in the NCBI (https://www.ncbi.nlm.nih.gov/) repository, accession number PRJNA645278.

## Author contributions

ZL and H-CW conceived and designed the experiment. ZL, XC, and Z-NG conducted the experiment and collected the samples. Z-NG uploaded the genomic data. W-HL and L-TC performed the analysis of the samples. ZL and J-RH analyzed the data. ZL wrote the first draft of the manuscript, which was later revised by H-CW, J-RH and C-HS. All authors contributed to the article and approved the submitted version.

## Supplementary material

Supplementary Figure 1 Phylogenetic tree generated from a maximum parsimony analysis based on the combined ITS, LSU, TUB and *rpb2* sequence alignment. Values above the branches represent parsimony bootstrap support values (>50%).

Supplementary Figure 2 Sample T41 gene length distribution. The horizontal axis denotes the length of the genes in base pairs, while the vertical axis represents the corresponding number of genes.

Supplementary Figure 3 Sample T41 gene functional annotation KEGG metabolic pathway classification map. The bars on the map correspond to the number of genes annotated for each functional class of level 1 in the KEGG database. The legend provides an interpretation of the code for each functional class.

Supplementary Figure 4 Sample T41 gene functional annotation KOG functional classification map. The x-axis of the plot represents the category of KOG (Clusters of Orthologous Groups) functional classification, while the y-axis represents the total number of genes annotated within each respective category.

Supplementary Figure 5 Sample T41 CAZymes functional classification and corresponding gene number statistics. The top row displays the sample ID, while the horizontal axis illustrates the CAZy database classification type, and the vertical axis represents the number of genes that have been annotated.

Supplementary Figure 6 Distribution of phenotypic mutation types in pathogens PHI. The x-axis represents the type of phenotypic mutation observed, while the y-axis indicates the number of genes annotated in the analysis.
