# Supplementary material for: Characteristics of Epicoccum latusicollum as revealed by genomic and metabolic phenomic analysis, the causal agent of tobacco Epicoccus leaf spot
Source: Front Plant Sci. 2023 Aug 24;14:1199956. doi: 10.3389/fpls.2023.1199956 (PMC10565823; doi:10.3389/fpls.2023.1199956)
Supplement: Supplementary file 1 [file DataSheet_1.docx]

**Supplementary table 1** Source of isolates and GenBank accession number in this study

|  |  |  | **GenBank Accession number** | | | |
| --- | --- | --- | --- | --- | --- | --- |
| **Species names** | **Strain no.** | **Original substrate** | **LSU** | **ITS** | ***rpb2*** | ***tub2*** |
| *E. brasiliense* | CBS 120105 | *Amaranthus sp* | GU238049 | GU237760 | KT389627 | GU237588 |
| *E. draconis* | CBS 186.83 | *Dracaena sp.* | GU238070 | GU237795 | KT389628 | GU237607 |
| *E. henningsii* | CBS 104.80 | *Acacia mearnsii* | GU238081 | GU237731 | KT389629 | GU237612 |
| *E. huancayense* | CBS 105.80 | *Solanum sp* | GU238084 | GU237732 | KT389630 | GU237615 |
| *E. nigrum* | CBS 125.82 | *Human toenail* | GU237974 | FJ426995 | KT389631 | FJ427106 |
| *E. plurivorum* | CBS 558.81 | *Setaria sp.* | GU238132 | GU237888 | KT389634 | GU237647 |
| *E. sorghinum* | CBS 179.80 | *Sorghum vulgare* | GU237978 | FJ427067 | KT389635 | FJ427173 |
| *E. latusicollum* | EP8 | *Elaeagnus pungens* | MT110968 | MT110966 | MT119152 | MT119151 |
| *E. sorghinum* | BPL01 | *Bothriochloa ischaemum* | OM453925 | OM453926 | OM863567 | OM451237 |
| *E. latusicollum* | T41 | *Nicotiana tabacum* | MN710367 | MN704804 | MN718013 | MN718012 |
| *E. layuense* | YHF0519 | *Oxalis corymbosa* | MN428044 | MN418006 | MZ437946 | MN428043 |
| *E. latusicollum* | JF-3 | *Zea mays* | OK445527 | OK483136 | OK490497 | OK490498 |
| *E. sorghinum* | GZDS2018BXT010 | *Camellia sinensis* | MK516207 | MK516206 | MK852278 | MK516208 |
| *E. latusicollum* | LC: 5158 | *Sorghum bicolor* | KY742255 | KY742101 | KY742174 | KY742343 |
| *E. sorghinum* | CBS 627.68 | *Citrus sp* | MH870912 | FJ427072 | KT389636 | FJ427178 |
| *E. latusicollum* | LC:4859 | *Camellia sinensis* | KY742256 | KY742102 | KY742175 | KY742344 |
| *E. latusicollum* | YC1105 | *Nicotiana tabacum* | MZ496641 | MZ496638 | MZ672002 | MZ672001 |

**Supplementary table 2** Substrates in PM 1-2 carbon source Micro plates significantly supported the growth of *Epicoccum latusicollum*

| **Well** | **Substrate** | **Well** | **Substrate** |
| --- | --- | --- | --- |
| **PM1** |  |  |  |
| A02 | *L*-Arabinose | D08 | *α*-Methyl-*D*-Galactoside |
| A10 | *D*-Trehalose | D09 | *α*-*D*-Lactose |
| A11 | *D*-Mannose | D10 | Lactulose |
| B08 | *D*-Xylose | D11 | Sucrose |
| B11 | *D*-Mannitol | E08 | *β*-Methyl-*D*-Glucoside |
| C04 | *D*-Ribose | E09 | Adonitol |
| C06 | *L*-Rhamnose | E10 | Maltotriose |
| C07 | *D*-Fructose | F11 | *D*-Cellobiose |
| C09 | *α*-*D*-Glucose | H02 | *p*-Hydroxy phenyl acetic acid |
| C10 | Maltose | H06 | *L*-Lyxose |
| C11 | *D*-Melibiose |  |  |
| **PM2** |  |  |  |
| A05 | *γ*-Cyclodextrin | B10 | *I*-Erythritol |
| A10 | Laminarin | C05 | Maltitol |
| B04 | Amygdalin | D04 | *L*-Sorbose |
| B05 | *D*-Arabinose | D07 | Turanose |
| B08 | Arbutin | H09 | Dihydroxy acetone |
| B09 | 2-Deoxy-*D*-Ribose |  |  |

**Supplementary table 3** Substrates in PM 3 nitrogen source Micro plates significantly supported the growth of *Epicoccum latusicollum*

| **Well** | **Substrate** | **Well** | **Substrate** | **Well** | **Substrate** |
| --- | --- | --- | --- | --- | --- |
| A03 | Nitrite | C03 | *D*-Alanine | F06 | Guanine |
| A04 | Nitrate | C04 | *D*-Asparagine | F07 | Guanosine |
| A07 | *L*-Alanine | C05 | *D*-Aspartic acid | F12 | Inosine |
| A08 | *L*-Arginine | C06 | *D*-Glutamic acid | G03 | Uric acid |
| A09 | *L*-Asparagine | C08 | *D*-Serine | G05 | Allantoin |
| A10 | *L*-Aspartic acid | C09 | *D*-Valine | G07 | *D*,*L*-*α*-Amino-*N*-Butyric acid |
| A11 | *L*-Cysteine | C10 | *L*-Citrulline | G08 | *γ*-Amino-*N*-Butyric acid |
| A12 | *L*-Glutamic acid | C11 | *L*-Homoserine | G10 | *D*,*L*-*α*-Amino-Caprylic acid |
| B01 | *L*-Glutamine | C12 | *L*-Ornithine | G11 | *δ*-Amino-*N*-Valeric acid |
| B02 | Glycine | D03 | *L*-Pyroglutamic acid | G12 | *α*-Amino-*N*-Valeric acid |
| B04 | *L*-Isoleucine | D06 | *N*-Amylamine | H01 | Ala-Asp |
| B05 | *L*-Leucine | D08 | Ethylamine | H02 | Ala-Gln |
| B06 | *L*-Lysine | D11 | Putrescine | H03 | Ala-Glu |
| B08 | *L-*Phenylalanine | D12 | Agmatine | H04 | Ala-Gly |
| B09 | *L*-Proline | E01 | Histamine | H05 | Ala-His |
| B10 | *L*-Serine | E02 | *β*-Phenylethyl amine | H08 | Gly-Asn |
| B11 | *L*-Threonine | E03 | Tyramine | H09 | Gly-Gln |
| B12 | *L*-Tryptophan | E11 | *N*-Acetyl-*D*-Glucosamine | H10 | Gly-Glu |
| C01 | *L*-Tyrosine | F02 | Adenine | H11 | Gly-Met |
| C02 | *L*-Valine | F03 | Adenosine | H12 | Met-Ala |

**Supplementary table 4** Substrates in PM 6-8nitrogen pathways Micro plates significantly supported the growth of *Epicoccum latusicollum*

| **Well** | **Substrate** | **Well** | **Substrate** | **Well** | **Substrate** | **Well** | **Substrate** |
| --- | --- | --- | --- | --- | --- | --- | --- |
| **PM6** |  |  |  |  |  |  |  |
| A02 | Positive control:*L*-Glutamine | B12 | Arg-Lys | D11 | Glu-Trp | G03 | Ile-Ala |
| A03 | Ala-Ala | C01 | Arg-Met | D12 | Glu-Tyr | G04 | Ile-Arg |
| A04 | Ala-Arg | C03 | Arg-Ser | E01 | Glu-Val | G05 | Ile-Gln |
| A05 | Ala-Asn | C05 | Arg-Tyr | E02 | Gly-Ala | G06 | Ile-Gly |
| A07 | Ala-Gly | C06 | Arg-Val | E05 | Gly-Gly | G08 | Ile-Ile |
| A08 | Ala-His | C07 | Asn-Glu | E06 | Gly-His | G10 | Ile-Phe |
| A09 | Ala-Leu | C08 | Asn-Val | E07 | Gly-Leu | G11 | Ile-Pro |
| A10 | Ala-Leu | C09 | Asp-Asp | E10 | Gly-Phe | G12 | Ile-Ser |
| A11 | Ala-Phe | D01 | Asp-Phe | F01 | Gly-Thr | H02 | Ile-Tyr |
| B01 | Ala-Ser | D02 | Asp-Trp | F02 | Gly-Trp | H03 | Ile-Val |
| B02 | Ala-Thr | D03 | Asp-Val | F03 | Gly-Tyr | H04 | Leu-Ala |
| B03 | Ala-Trp | D04 | Cys-Gly | F04 | Gly-Val | H05 | Leu-Arg |
| B05 | Arg-Ala | D05 | Gln-Gln | F05 | His-Asp | H06 | Leu-Asp |
| B06 | Arg-Arg | D06 | Gln-Gly | F06 | His-Gly | H07 | Leu-Glu |
| B07 | Arg-Asp | D07 | Glu-Asp | F10 | His-Pro | H08 | Leu-Gly |
| B09 | Arg-Glu | D08 | Glu-Glu | F11 | His-Ser | H09 | Leu-Ile |
| B10 | Arg-Ile | D09 | Glu-Gly | F12 | His-Trp | H12 | Leu-Phe |
| B11 | Arg-Leu | D10 | Glu-Ser | G02 | His-Val |  |  |
| **PM7** |  |  |  |  |  |  |  |
| A02 | Positive control:*L*-Glutamine | C11 | Phe-Ile | E11 | Thr-Ala | G06 | Tyr-Gln |
| A03 | Leu-Ser | C12 | Phe-Phe | E12 | Thr-Arg | G08 | Tyr-Gly |
| A04 | Leu-Trp | D01 | Phe-Pro | F01 | Thr-Glu | G09 | Tyr-His |
| A05 | Leu-Val | D03 | Phe-Trp | F02 | Thr-Gly | G10 | Tyr-Leu |
| A06 | Lys-Ala | D04 | Pro-Ala | F03 | Thr-Leu | G11 | Tyr-Lys |
| A07 | Lys-Arg | D05 | Pro-Asp | F04 | Thr-Met | G12 | Tyr-Phe |
| A10 | Lys-Leu | D06 | Pro-Gln | F05 | Thr-Pro | H01 | Tyr-Trp |
| A11 | Lys-Lys | D07 | Pro-Gly | F06 | Trp-Ala | H02 | Tyr-Tyr |
| A12 | Lys-Phe | D08 | Pro-Hyp | F07 | Trp-Arg | H03 | Val-Arg |
| B01 | Lys-Pro | D09 | Pro-Leu | F08 | Trp-Asp | H04 | Val-Asn |
| B02 | Lys-Ser | D10 | Pro-Phe | F09 | Trp-Glu | H05 | Val-Gly |
| B03 | Lys-Thr | D12 | Pro-Tyr | F10 | Trp-Gly | H06 | Val-Gly |
| B04 | Lys-Trp | E01 | Ser-Ala | F11 | Trp-Leu | H08 | Val-Ile |
| B05 | Lys-Tyr | E03 | Ser-His | F12 | Trp-Lys | H09 | Val-Leu |
| B06 | Lys-Val | E04 | Ser-Leu | G01 | Trp-Phe | H10 | Val-Tyr |
| B09 | Met-Gln | E06 | Ser-Phe | G02 | Trp-Ser | H11 | Val-Val |
| C07 | Met-Trp | E07 | Ser-Pro | G03 | Trp-Trp |  |  |
| C09 | Phe-Ala | E08 | Ser-Ser | G04 | Trp-Tyr |  |  |
| C10 | Phe-Gly | E10 | Ser-Val | G05 | Tyr-Ala |  |  |
| **PM8** |  |  |  |  |  |  |  |
| A02 | Positive Control:*L*-Glutamine | B12 | Lys-Asp | D08 | Ser-Asp | F09 | *D-*Ala-Leu |
| A03 | Ala-Asp | C01 | Lys-Gly | D09 | Ser-Gln | F12 | *D*-Leu-Tyr |
| A04 | Ala-Gln | C03 | Met-Phr | D10 | Ser-Glu | G01 | *γ*-Glu-Gly |
| A05 | Ala-lle | C04 | Met-Tyr | D11 | Thr-Asp | G05 | Gly-*D*-Ser |
| A07 | Ala-Val | C05 | Phe-Asp | D12 | Thr-Gln | G08 | Leu-*β*-Ala |
| A08 | Asp-Ala | C06 | Phe-Glu | E01 | Thr-Phe | G10 | Phe-*β*-Ala |
| A09 | Asp-Gln | C07 | Gln-Glu | E03 | Trp-Val | G11 | Ala-Ala-Ala |
| A10 | Ala-Gly | C09 | Phe-Tyr | E04 | Tyr-lle | H01 | Gly-Gly-Ala |
| A12 | Gly-Asn | C10 | Phe-Val | E05 | Tyr-Val | H03 | Gly-Gly-Gly |
| B01 | Gly-Asp | C11 | Pro-Arg | E06 | Val-Ala | H05 | Gly-Gly-Leu |
| B02 | Gly-lle | C12 | Pro-Asn | E07 | Val-Gln | H06 | Gly-Gly-Phe |
| B03 | His-Ala | D01 | Pro-Glu | E08 | Val-Glu | H07 | Val-Tyr-Val |
| B04 | His-Glu | D02 | Pro-lle | E09 | Val-Lys | H08 | Gly-Phe-Phe |
| B06 | lle-Asn | D03 | Pro-Lys | E11 | Val-Phe | H09 | Leu-Gly-Gly |
| B07 | Ile-Leu | D04 | Pro-Ser | E12 | Val-Pro | H11 | Phe-Gly-Gly |
| B08 | Leu-Asn | D05 | Pro-Trp | F01 | Val-Ser | H12 | Tyr-Gly-Gly |
| B10 | Leu-Pro | D06 | Pro-Val | F03 | *β*-Ala-Gly |  |  |
| B11 | Leu-Tyr | D07 | Ser-Asn | F05 | Met-*β*-Ala |  |  |

**Supplementary table 5** Substrates in PM 4 phosphorus and sulfur source Micro plates significantly supported the growth of *Epicoccum latusicollum*

| **Well** | **Substrate** |
| --- | --- |
| A12 | Adenosine-3',5'-Cyclic monophosphate |
| C07 | 6-Phospho-Gluconic acid |
| C12 | Cytidine- 3',5'-Cyclic monophosphate |
| E11 | Inositol hexaphosphate |
| G12 | *L*-Methionine sulfone |
| H01 | *L*-Djenkolic acid |
| H04 | *D,L*-Lipoamide |
| H10 | 2-Hydroxyethane sulfonic acid |
| H11 | Methane sulfonic acid |
| H12 | Tetramethylene sulfone |

**Supplementary table 6** Substrates in PM 9-10 osmotic and ionic conditions, and pH environments Micro plates significantly supported the growth of *Epicoccum latusicollum*

| **Well** | **Substrate** | **Well** | **Substrate** | **Well** | **Substrate** |
| --- | --- | --- | --- | --- | --- |
| **PM9** |  |  |  |  |  |
| A01 | NaCl 1% | C05 | NaC1 6% +*γ-*Amino-n-butyric acid | F02 | Sodium Lactate 2% |
| A02 | NaCl 2% | C06 | NaC1 6% + Glutathione | F03 | Sodium Lactate 3% |
| A03 | NaCl 3% | C07 | NaCl 6% + Glycerol | F04 | Sodium Lactate 4% |
| A04 | NaCl 4% | C08 | NaCl 6% + Trehalose | F05 | Sodium Lactate 5% |
| A05 | NaCl 5% | C09 | NaC1 6% + Trimethylamine-*N*-oxide | F06 | Sodium Lactate 6% |
| A06 | NaCl 5.5% | C10 | NaC1 6%+ Trimethylamine | F07 | Sodium Lactate 7% |
| A07 | NaCl 6% | C11 | NaC1 6% + Octopine | F08 | Sodium Lactate 8% |
| A08 | NaCl 6.5% | C12 | NaC1 6% + Trigonelline | F09 | Sodium Lactate 9% |
| A09 | NaCl 7% | D01 | Potassium chloride 3% | F10 | Sodium Lactate 10% |
| A10 | NaCl 8% | D02 | Potassium chloride 4% | F11 | Sodium Lactate 11% |
| A11 | NaCl 9% | D03 | Potassium chloride 5% | F12 | Sodium Lactate 12% |
| A12 | NaCl 10% | D04 | Potassium chloride 6% | G01 | Sodium Phosphate pH7 20mM |
| B01 | NaCl 6% | D05 | Sodium sulfate 2% | G02 | Sodium Phosphate pH7 50mM |
| B02 | NaCl 6% +Betaine | D06 | Sodium sulfate 3% | G03 | Sodium Phosphate pH7 100mM |
| B03 | NaCl 6% +*N-N*Dimethylglycine | D07 | Sodium sulfate 4% | G04 | Sodium Phosphate pH7 200mM |
| B04 | NaCl 6% + Sarcosine | D08 | Sodium sulfate 5% | G09 | Ammonium sulfate pH8 10mM |
| B05 | NaCl 6% + Dimethyl sulphonyl propionate | D09 | Ethylene glycol5% | G10 | Ammonium sulfate pH8 20mM |
| B06 | NaCl 6% + MOPS | D10 | Ethylene glycol10% | G11 | Ammonium sulfate pH8 50mM |
| B07 | NaCl 6% + Ectoine | D11 | Ethylene glycol15% | G12 | Ammonium sulfate pH8 100mM |
| B08 | NaCl 6% + Choline | D12 | Ethylene glycol20% | H01 | Sodium Nitrite 10mM |
| B09 | NaCl 6% + Phosphorylcholine | E01 | Sodium formate 1% | H02 | Sodium Nitrate 20 mM |
| B10 | NaCl 6% + Creatine | E02 | Sodium formate 2% | H03 | Sodium Nitrate 40 mM |
| B11 | NaCl 6% + Creatinine | E03 | Sodium formate 3% | H04 | Sodium Nitrite 60mM |
| B12 | NaCl 6% + *L*-Carnitine | E04 | Sodium formate 4% | H05 | Sodium Nitrate 80 mM |
| C01 | NaC1 6% +KCl | E05 | Sodium formate 5% | H06 | Sodium Nitrate 100 mM |
| C02 | NaCl 6%+*L*-proline | E06 | Sodium formate 6% | H07 | Sodium Nitrite 10mM |
| C03 | NaCl 6%+*N*-Acethyl-*L*-glutamine | E07 | Urea 2% |  |  |
| C04 | NaCl 6% +*β*-Glutamic acid | F01 | Sodium Lactate 1% |  |  |
| **PM10** |  |  |  |  |  |
| A01 | pH 3.5 | C04 | pH 4.5+*L*-Serine | F04 | pH 9.5+*L*-Serine |
| A02 | pH 4 | C05 | pH 4.5+*L*-Threonine | F06 | pH 9.5+*L*-Tryptophan |
| A03 | pH 4.5 | C06 | pH 4.5+ *L*-Tryptophan | F07 | pH 9.5+ *L*-Tyrosine |
| A04 | pH 5 | C07 | pH 4.5+*L*-Tyrosine | F10 | pH 9.5+*L*-Ornithine |
| A05 | pH5.5 | C08 | pH 4.5+*L*-Valine | F12 | pH 9.5+ *L*-Homoserine |
| A06 | pH 6 | C09 | pH 4.5+ Hydroxy-*L*-Proline | G01 | pH 9.5+ Anthranilic acid |
| A07 | pH 7 | C10 | pH 4.5+*L*-Ornithine | G02 | pH 9.5+*L*-Norleucine |
| A08 | pH 8 | C11 | pH 4.5+*L*-Homoarginine | G04 | pH 9.5+ Agmatine |
| A09 | pH 8.5 | C12 | pH 4.5+*L*-Homoserine | G05 | pH 9.5+ Cadaverine |
| A10 | pH 9 | D02 | pH 4.5+*L*-Norleucine | G06 | pH 9.5+ Putrescine |
| A11 | pH 9.5 | D03 | pH 4.5+*L*-Norvaline | G09 | pH 9.5+ Tyramine |
| A12 | pH 10 | D04 | pH 4.5+ *α-*Amino-*N*-Butyric acid | G10 | pH 9.5+ Creatine |
| B01 | pH 4.5 | D06 | pH 4.5+ *L*-Cysteic acid | G11 | pH 9.5+ Trimethylamine-*N*-oxide |
| B02 | pH 4.5+*L*-Alanine | D07 | pH 4.5+*D*-Lysine | H01 | *X*-Caprylate |
| B03 | pH4.5+*L*-Argine | D08 | pH 4.5+ 5-Hydroxy Lysine | H02 | *X-α-D*-Glucoside |
| B04 | *L*-Asparagine | D09 | pH 4.5 + 5-Hydroxy tryptophan | H03 | *X-β-D*-Glucoside |
| B05 | pH 4.5+*L*-Aspartic acid | D10 | pH 4.5+ *D*,*L*-Diaminopimelic acid | H04 | *X-α-D*-Galactoside |
| B06 | pH 4.5+ *L*-Glutamic acid | D11 | pH 4.5+ Trimethylamine-*N*-oxide | H05 | *X-β-D*-Galactoside |
| B07 | pH 4.5+*L*-Glutamine | D12 | pH 4.5+ Urea | H06 | *X-α-D-*Glucuronide |
| B08 | pH 4.5+ Glycine | E01 | pH 9.5 | H07 | *X-β-D*-GIucuronide |
| B09 | pH 4.5+ *L*-Histidine | E03 | pH 9.5+*L*-Arginine | H08 | *X-β-D*-Glucosaminide |
| B10 | pH 4.5+*L*-Isoleucine | E11 | pH 9.5+*L*-Leucine | H09 | *X*-*β*-*D*-Galactosaminide |
| B12 | pH 4.5+*L*-Lysine | E12 | pH 9.5+*L*-Lysine | H10 | *X-α-D-*Mannoside |
| C01 | pH 4.5+*L*-Methionine | F01 | pH 9.5+*L*-Methionine | H11 | *X*-PO4 |
| C02 | pH 4.5+*L*-Phenylalanine | F02 | pH 9.5+*L*-Phenylalanine | H12 | *X*-SO4 |
| C03 | pH 4.5+*L*-Proline | F03 | pH 9.5+*L*-Proline |  |  |

**Supplementary table 7** The effect of *Epicoccum latusicollum* on metabolism of tobacco phyllosphere microorganism

| Carbon sources | T41 samples of healthy | T41 samples of diseased |
| --- | --- | --- |
| *β*-Methyl-D-Glucoside | +++ | + |
| *D*-Galactonic Acid y-Lactone | +++ | + |
| *L*-Arginine | +++ | + |
| Pyruvic Acid Methyl Ester | +++ | + |
| *D*-Xylose | ++ | + |
| *D*-Galacturonic Acid | +++ | + |
| *L*-Asparagine | +++ | + |
| Tween 40 | +++ | ++ |
| I-Erythritol | +++ | + |
| 2-Hydroxy Benzoic Acid | ++ | + |
| *L*-Phenylalanine | ++ | + |
| Tween 80 | +++ | + |
| *D*-Mannitol | +++ | + |
| 4-Hydroxy Benzoic Acid | + | + |
| *L*-Serine | +++ | + |
| *α*-Cyclodextrin | +++ | + |
| *N*-Acetyl-D-Glucosamine | +++ | + |
| *γ*-Hydroxybutyric Acid | +++ | + |
| *L*-Threonine | ++ | + |
| Glycogen | +++ | + |
| *D*-Glucosaminic Acid | +++ | + |
| Itaconic Acid | +++ | + |
| Glycyl-L-Glutamic Acid | +++ | + |
| *D*-Cellobiose | +++ | + |
| Glucose-1-Phosphate | +++ | + |
| *α*-Ketobutyric Acid | + | + |
| Phenylethyl-amine | +++ | + |
| *α*-D-Lactose | +++ | + |
| *D*,L-a-Glycerol | +++ | + |
| *D*-Malic Acid | +++ | + |
| Putrescine | ++ | + |
