# Supplementary figures and images for: Characteristics of Epicoccum latusicollum as revealed by genomic and metabolic phenomic analysis, the causal agent of tobacco Epicoccus leaf spot
Source: Front Plant Sci. 2023 Aug 24;14:1199956. doi: 10.3389/fpls.2023.1199956 (PMC10565823; doi:10.3389/fpls.2023.1199956)

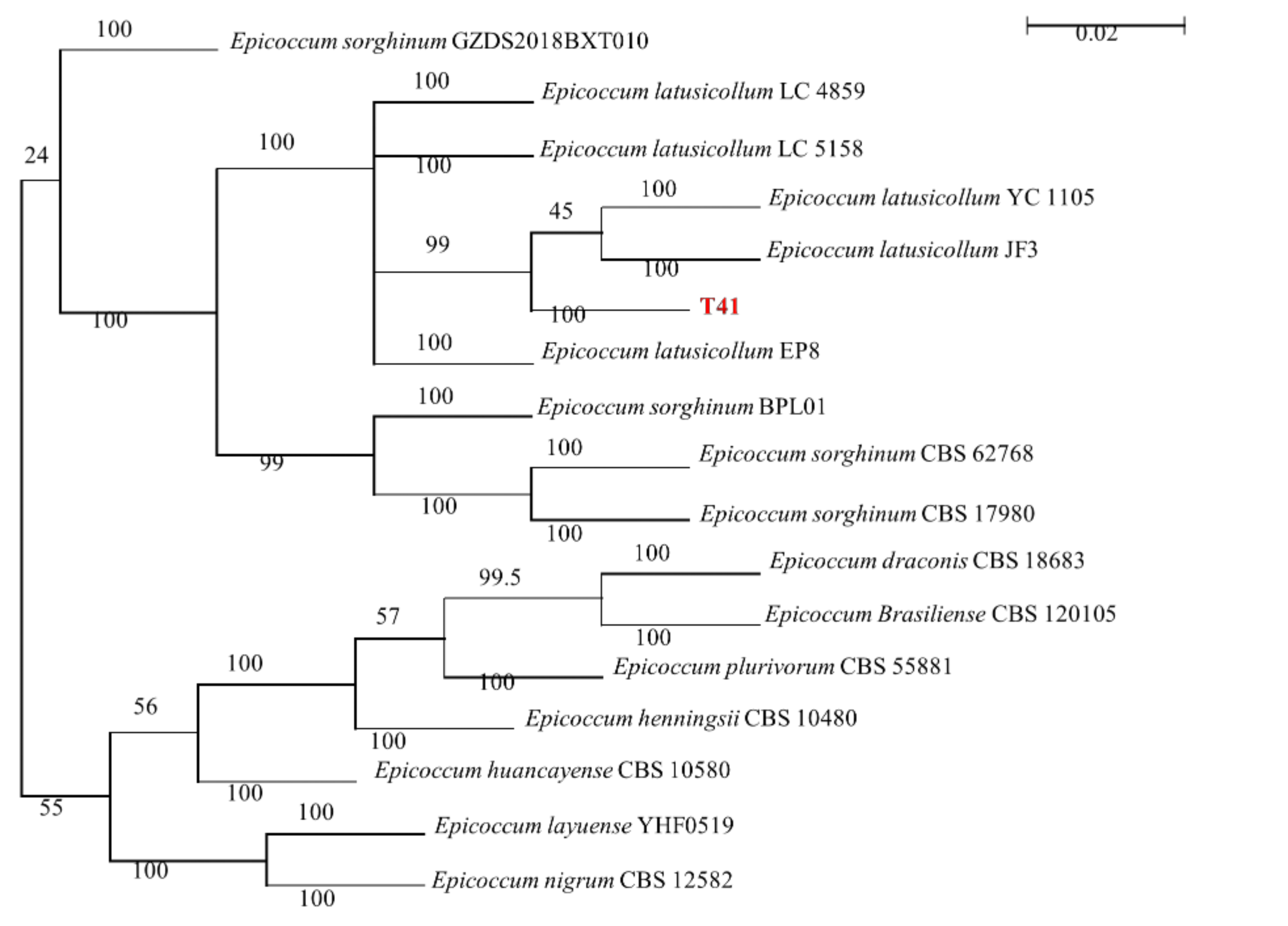

Supplement: Supplementary Figure 1 — Phylogenetic tree generated from a maximum parsimony analysis based on the combined ITS, LSU, TUB and rpb2 sequence alignment. Values above the branches represent parsimony bootstrap support values (>50%). [file Image_1.jpeg]

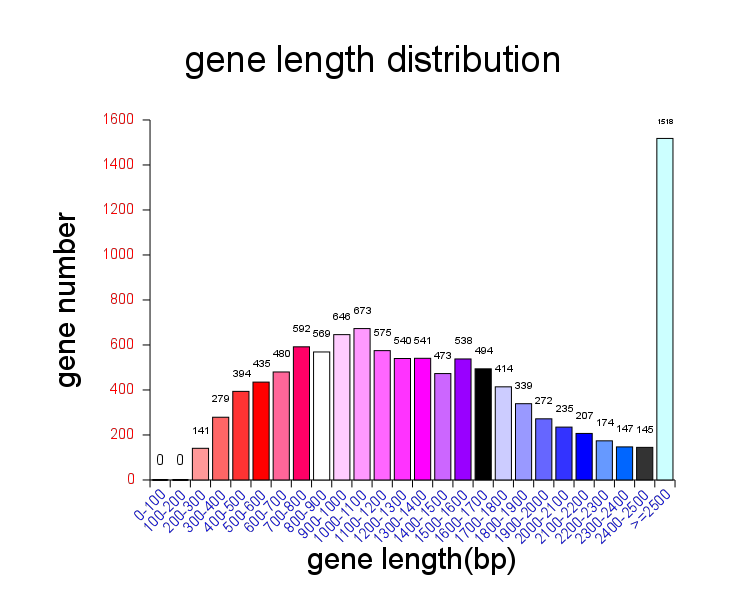

Supplement: Supplementary Figure 2 — Sample T41 gene length distribution. The horizontal axis denotes the length of the genes in base pairs, while the vertical axis represents the corresponding number of genes. [file Image_2.tif]

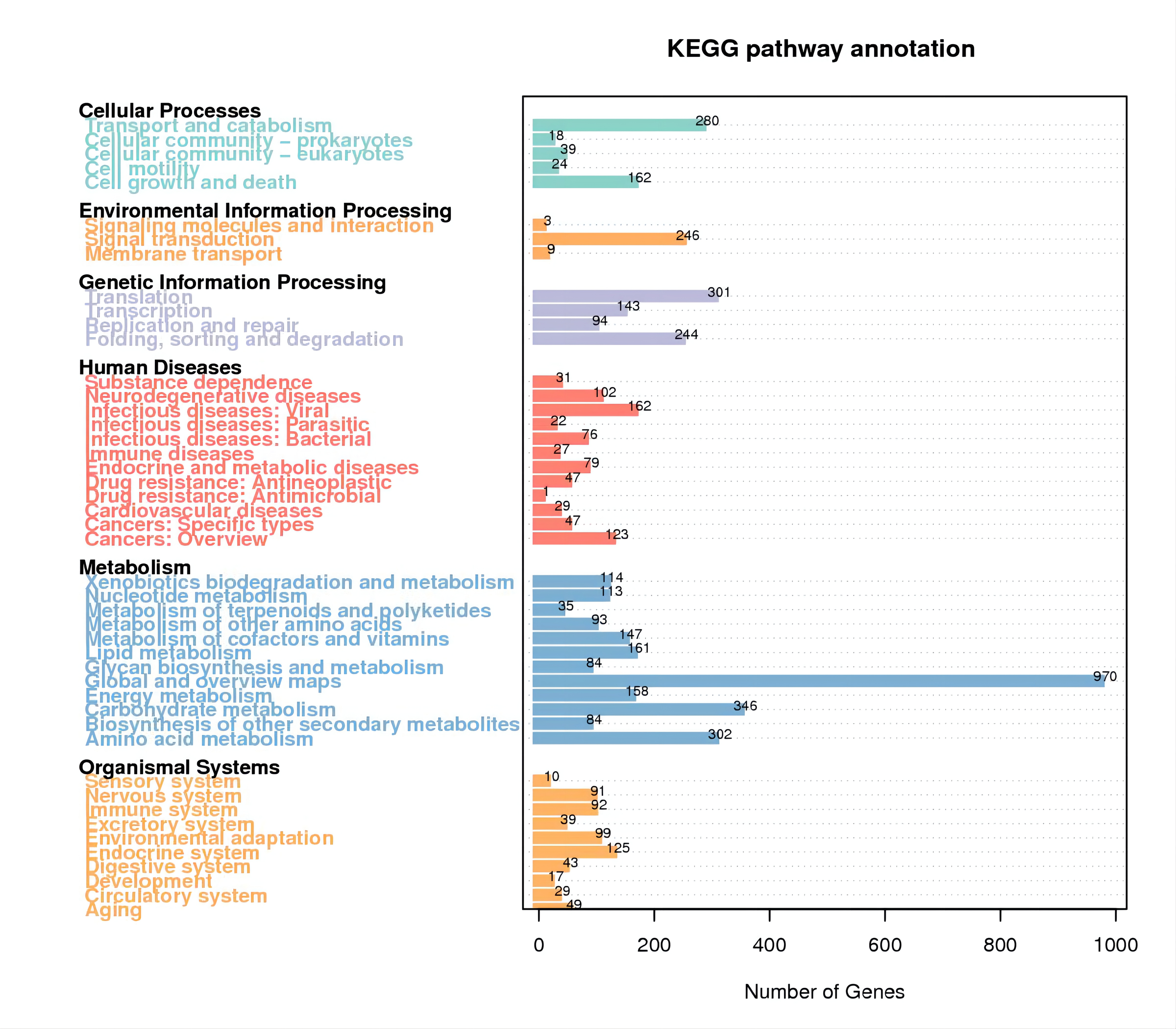

Supplement: Supplementary Figure 3 — Sample T41 gene functional annotation KEGG metabolic pathway classification map. The bars on the map correspond to the number of genes annotated for each functional class of level 1 in the KEGG database. The legend provides an interpretation of the code for each functional class. [file Image_3.tif]

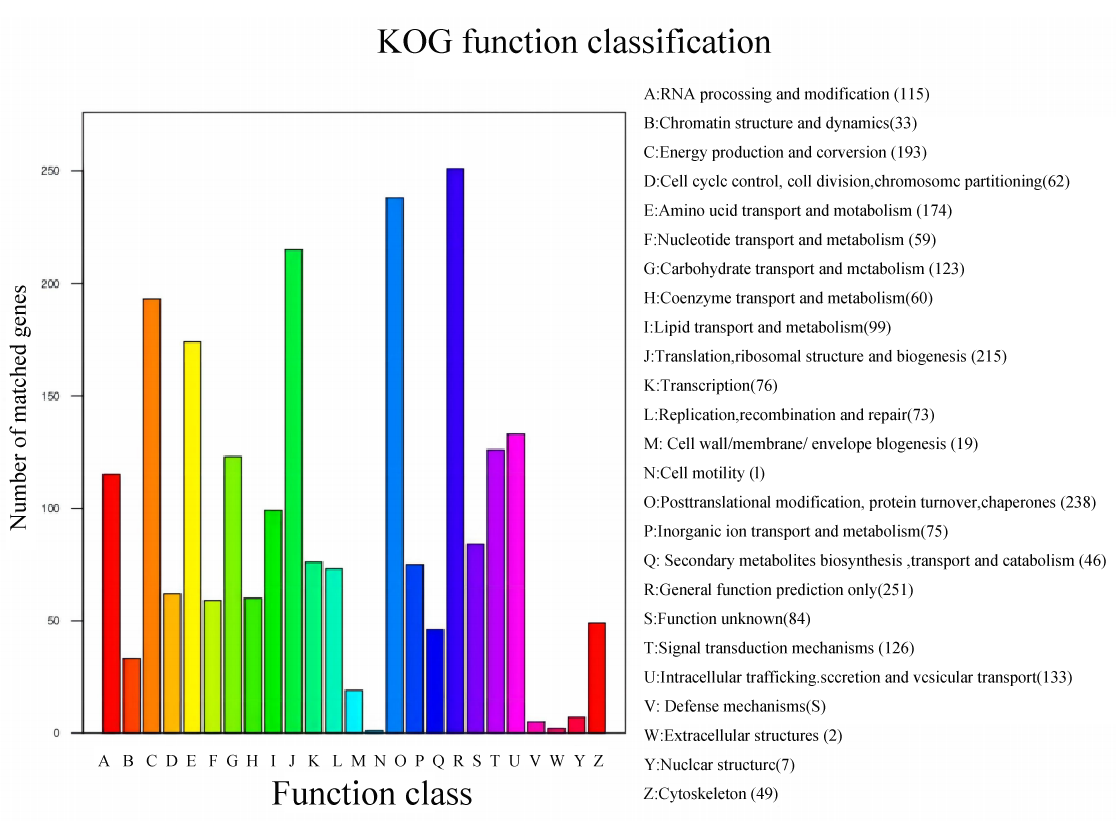

Supplement: Supplementary Figure 4 — Sample T41 gene functional annotation KOG functional classification map. The x-axis of the plot represents the category of KOG (Clusters of Orthologous Groups) functional classification, while the y-axis represents the total number of genes annotated within each respective category. [file Image_4.tif]

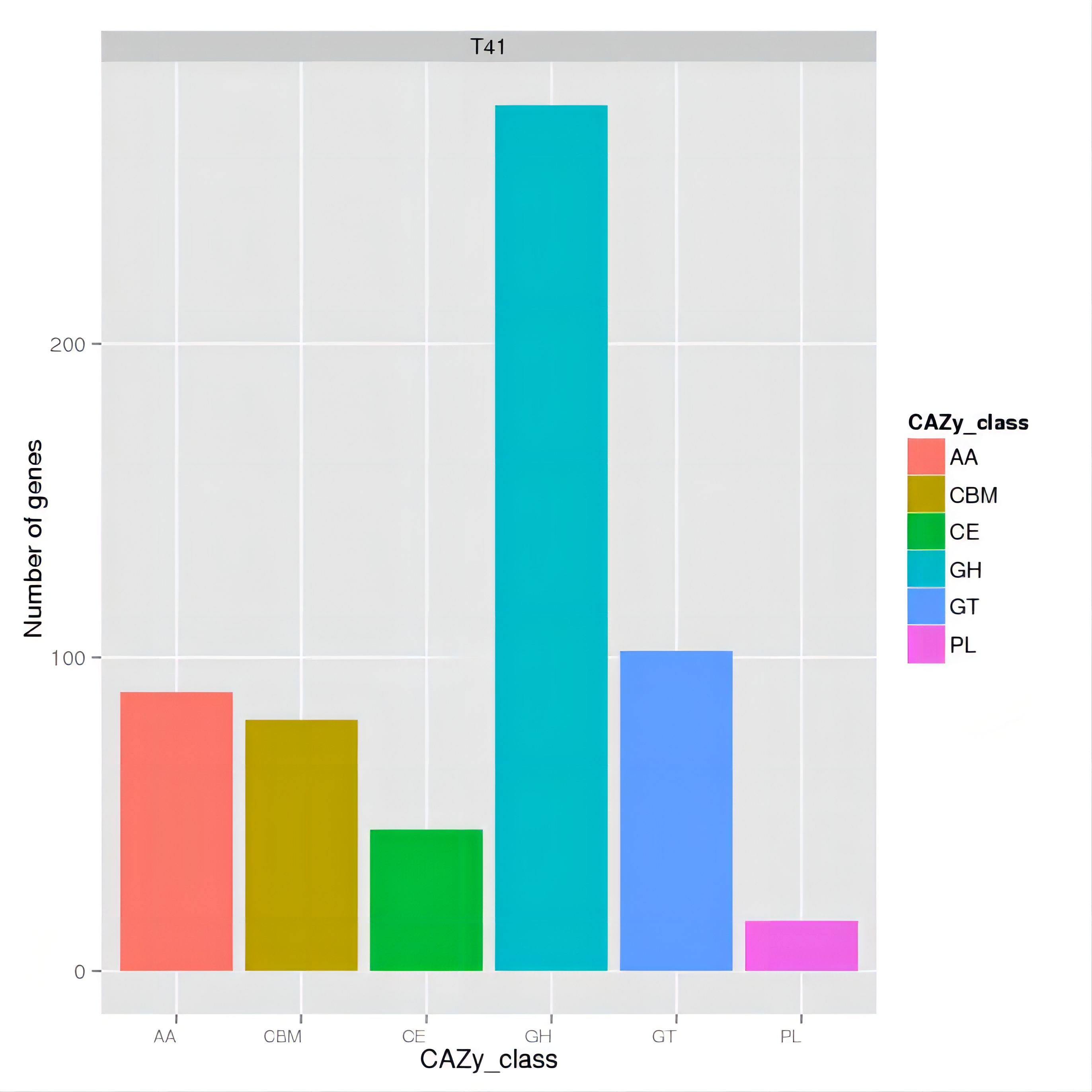

Supplement: Supplementary Figure 5 — Sample T41 CAZymes functional classification and corresponding gene number statistics. The top row displays the sample ID, while the horizontal axis illustrates the CAZy database classification type, and the vertical axis represents the number of genes that have been annotated. [file Image_5.tif]

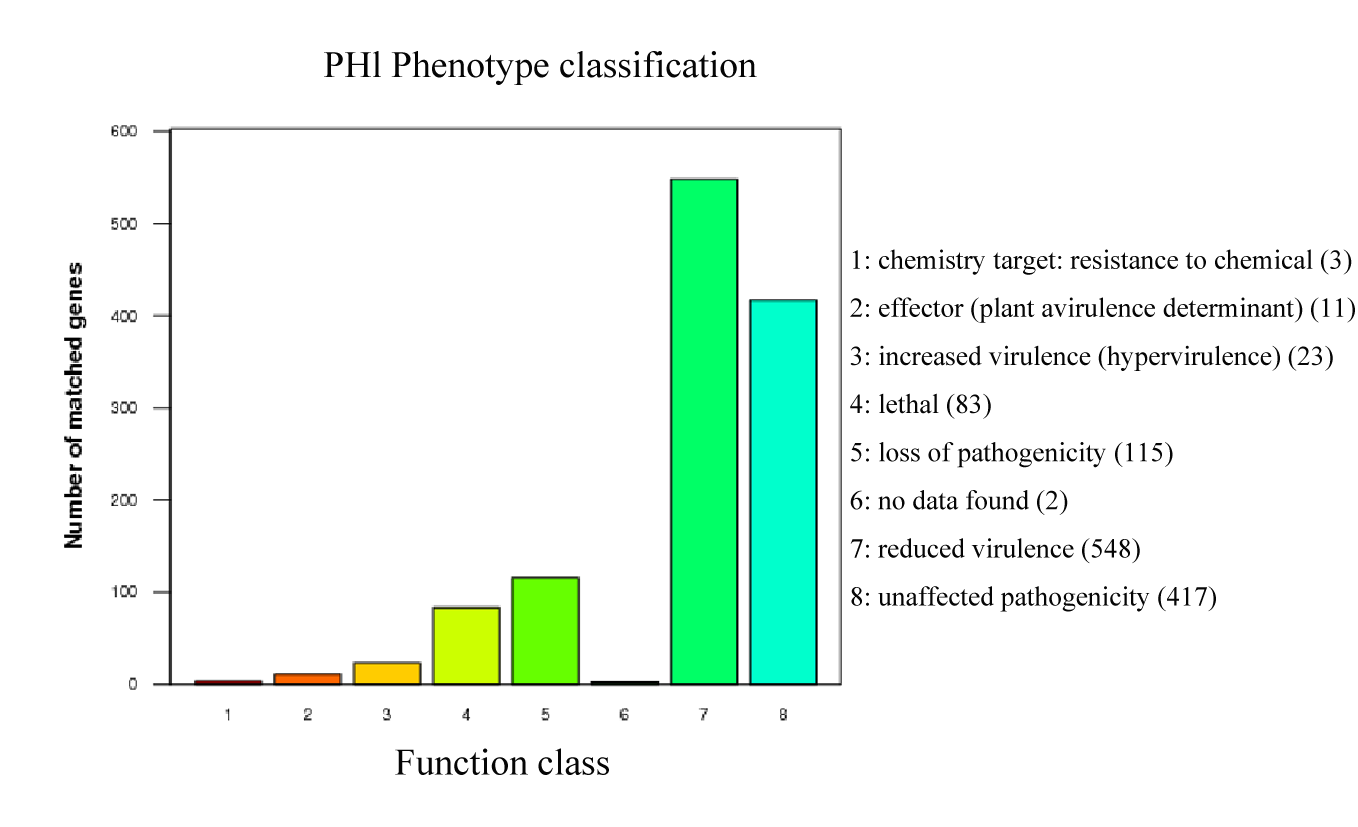

Supplement: Supplementary Figure 6 — Distribution of phenotypic mutation types in pathogens PHI. The x-axis represents the type of phenotypic mutation observed, while the y-axis indicates the number of genes annotated in the analysis. [file Image_6.tif]
